# Functional Characterization of Ubiquitin-Like Core Autophagy Protein ATG12 in *Dictyostelium discoideum*

**DOI:** 10.3390/cells8010072

**Published:** 2019-01-19

**Authors:** Sarah Fischer, Ramesh Rijal, Peter Frommolt, Prerana Wagle, Roman Konertz, Jan Faix, Susanne Meßling, Ludwig Eichinger

**Affiliations:** 1 Institute of Biochemistry I, Medical Faculty, University Hospital Cologne, 50931 Cologne, Germany; sarah.fischer@uni-koeln.de (S.F.); rkonertz@uni-koeln.de (R.K.); susanne_messling@web.de (S.M.); 2 Department of Biology, Texas A & M University, Laredo, TX 77843-3474, USA; rrijal@bio.tamu.edu; 3 Bioinformatics Core Facility, CECAD Research Center, University Hospital Cologne, 50931 Cologne, Germany; peter.frommolt@t-online.de (P.F.); prerana.wagle@uk-koeln.de (P.W.); 4 Institute for Biophysical Chemistry, Hannover Medical School, 30625 Hannover, Germany; faix.jan@mh-hannover.de

**Keywords:** ATG12, ATG16, autophagy, *Dictyostelium*, ubiquitin-like protein, post-translational modifier, phagocytosis, pinocytosis, proteasome, ubiquitin proteasome system (UPS)

## Abstract

Autophagy is a highly conserved intracellular degradative pathway that is crucial for cellular homeostasis. During autophagy, the core autophagy protein ATG12 plays, together with ATG5 and ATG16, an essential role in the expansion of the autophagosomal membrane. In this study we analyzed gene replacement mutants of *atg12* in *Dictyostelium discoideum* AX2 wild-type and ATG16‾ cells. RNA_seq_ analysis revealed a strong enrichment of, firstly, autophagy genes among the up-regulated genes and, secondly, genes implicated in cell motility and phagocytosis among the down-regulated genes in the generated ATG12‾, ATG16‾ and ATG12‾/16‾ cells. The mutant strains showed similar defects in fruiting body formation, autolysosome maturation, and cellular viability, implying that ATG12 and ATG16 act as a functional unit in canonical autophagy. In contrast, ablation of ATG16 or of ATG12 and ATG16 resulted in slightly more severe defects in axenic growth, macropinocytosis, and protein homeostasis than ablation of only ATG12, suggesting that ATG16 fulfils an additional function in these processes. Phagocytosis of yeast, spore viability, and maximal cell density were much more affected in ATG12‾/16‾ cells, indicating that both proteins also have cellular functions independent of each other. In summary, we show that ATG12 and ATG16 fulfil autophagy-independent functions in addition to their role in canonical autophagy.

## 1. Introduction

Macroautophagy, hereafter denoted as autophagy for simplicity, is the major lysosomal route for the clearance and turnover of damaged organelles and long-lived proteins [1]. It occurs at basal levels in most cell types and is induced in response to cellular stresses such as starvation, the presence of protein aggregates, and invading pathogens [2,3]. Most likely, autophagy evolved in unicellular organisms as a survival mechanism during starvation through the recycling of cellular constituents [4,5]. The basic molecular machinery works in a sequential order to deliver cytoplasmic cargo to the lysosome and was initially discovered in the budding yeast *Saccharomyces cerevisiae* [6]. The proteins involved in autophagosome formation were named ATG, for AuTophaGy-related proteins, and are evolutionarily highly conserved across the eukaryotic lineage [7,8]. Autophagic dysfunction can result in a wide range of diseases, including neurodegeneration, cancer, muscular dystrophy, and lipid-storage disorders [3,9].

**Figure 1:**
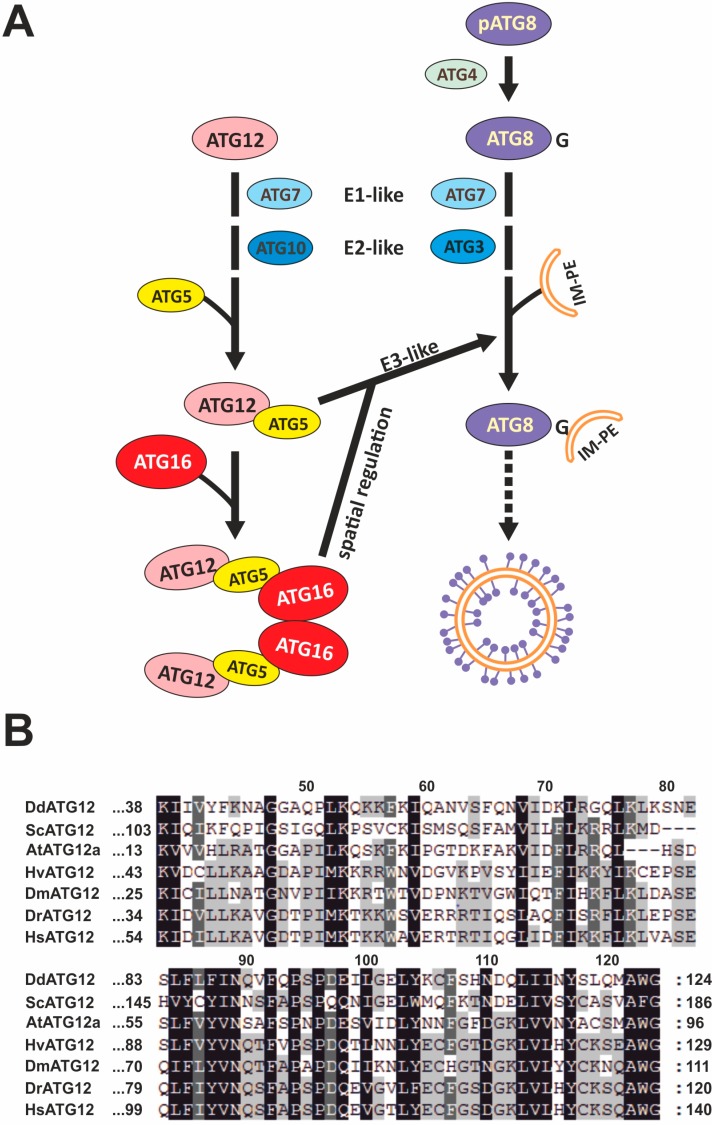
The two ubiquitin-like conjugation systems and multiple sequence alignment of the APG12 domain. (**A**) Schematic representation of the components and their interrelations of the two ubiquitin-like conjugation systems in autophagy. The ATG12 (left) and the ATG8/LC3 (right) conjugation systems are depicted. The different components of the two conjugation systems are not drawn to scale. Legend: pATG8, proATG8; IM-PE, isolation membrane containing phosphatidylethanolamine. Modified from Pyo et al. [30]. See text for further details. (**B**) Multiple sequence alignment of ATG12 orthologs. The highly conserved APG12 domain of ATG12 from different organisms is shown. The multiple sequence alignment was performed with Clustal Omega [31] and displayed using the Genedoc program (v. 2.7). Amino acid residues are numbered and sequence similarity is indicated by shading. Conserved percent: dark grey = 100%, medium grey = 80%, light grey = 60%. Legend: Dd, *Dictyostelium discoideum*; Sc, *Saccharomyces cerevisiae*; At, *Arabidopsis thaliana*; Hv, *Hydra vulgaris*; Dm, *Drosophila melanogaster*; Dr, *Danio rerio*; Hs, *Homo sapiens*.

The autophagic process can be subdivided into initiation, maturation, and lysosomal degradation phases. In the initiation phase, the so-called omegasome (phagophore assembly site or PAS in *S. cerevisiae*), a specialized subdomain of the endoplasmatic reticulum (ER), is generated de novo, and then becomes the isolation membrane in higher eukaryotes or the phagophore in yeast. This structure further expands through the incorporation of membrane lipids, engulfs entire organelles or parts of the cytoplasm and finally closes into a double-membrane structure, the autophagosome [1,10]. Autophagosomes eventually mature into autolysosomes upon fusion of the outer autophagosomal membrane with the lysosomal membrane. Finally, the inner autophagosomal membrane and the cargo are degraded by lysosomal hydrolases [5]. Two ubiquitin-like conjugation systems are indispensable for the expansion of the isolation membrane [11]. Similarly to the ubiquitin system, the two ubiquitin-like proteins ATG12 and ATG8 (LC3 in mammals) are finally attached to their substrate via this enzymatic pathway. In the first ubiquitin-like reaction, ATG12 is activated by the E1-like enzyme ATG7 and then conjugated to the E2-like enzyme ATG10 [12,13]. Subsequently, ATG12 is covalently attached to its target protein ATG5, and two ATG12~5 conjugates in turn associate non-covalently with an ATG16 dimer [14,15] (Figure 1A). The ATG12~5 conjugation seems to be irreversible, since so far no enzyme for the cleavage of the isopeptide bond between ATG12 and ATG5 has been identified [11]. In the final step of the second ubiquitin-like reaction, ATG8 is reversibly attached to the lipid phosphatidylethanolamine (PE) on the expanding autophagosomal membrane via the E3-like activity of the ATG12~5/16 complex. It is believed that the complex brings the ATG8-carrying E2-like enzyme ATG3 in proximity to PE and determines the exact site of ATG8-PE production on the autophagosomal membrane [16,17,18] (Figure 1A).

The ubiquitin-like protein ATG12 was originally discovered in *S. cerevisiae* [6]. Its 3D structure is similar to the structure of ubiquitin and is highly conserved from yeast to man. ATG12 proteins from different organisms share a so-called APG12 domain which shows the conserved ubiquitin-fold in the crystal structure [11] (Figure 1B). The APG12 domain is required for both the conjugation to ATG5 and canonical autophagy [19]. ATG12 is part of the heterotetrameric ATG12~5/16 complex which localizes to the outer membrane of the expanding isolation membrane and is released shortly before or after autophagosome completion [20]. The association of the ATG12~5 conjugate with ATG16 unmasks a membrane-binding site in ATG5 and the membrane tethering ability of ATG5 is also stimulated by ATG12 [18]. Within the ATG12~5/16 complex, ATG16 is required for correct localization and the ATG12~5 conjugate possesses E3 ligase activity that promotes the conjugation of ATG8 to PE at the autophagic membrane [17,21,22]. Knock-out mutants of ATG12 have shown postnatal lethality in mice and are not able to form cysts and fruiting bodies in Ascomyceta and Amoebozoa [23,24,25,26]. However, despite extensive study, the precise cellular functions of ATG12 are still not fully understood.

The social amoeba *D. discoideum* is a well-established model organism used to study the autophagic process [27]. Under nutrient-rich conditions, *Dictyostelium* cells grow as unicellular amoebae that divide by binary cell fission and feed on bacteria by phagocytosis [28]. Upon depletion of the food source, solitary amoebae aggregate and undergo distinct morphological states, giving rise to mature fruiting bodies [29]. Since the developmental stage takes place in the absence of nutrients, *Dictyostelium* cells mobilize a large fraction of the required energy for morphogenesis and biosynthetic pathways by autophagy [27].

Here we describe the consequences of the deletion of *atg12* in AX2 wild-type and ATG16‾ cells for genome-wide transcription, development, autolysosome formation, growth, phagocytosis, macropinocytosis, and protein homeostasis. Our results reveal massive transcriptional changes and complex phenotypes of varying severity for the different knock-out strains, implying that ATG12 and ATG16 have, in addition to their role in canonical autophagy, autophagy-independent functions. Moreover, we could detect ATG12 only in the ATG12~5 conjugate and found no evidence for unconjugated ATG12. Our results also support links between autophagy and the uptake of nutrients as well as between autophagy and the ubiquitin-proteasome system (UPS).

## 2. Materials and Methods

### 2.1. Dictyostelium Strains, Growth, and Development

*D. discoideum* AX2 was used as wild-type strain. The ATG12‾ and ATG12‾/16‾ strains were generated by replacement of the *atg12* gene with the knock-out construct in AX2 and ATG16‾ cells [32]. Strains expressing RFP-ATG12 or RFP-GFP-ATG8a were generated by transformation of AX2 and knock-out strains, respectively, with appropriate expression constructs as described below. The strains used in this study are listed in Table 1. All *D. discoideum* strains were grown at 22 °C in liquid nutrient medium on plates (10 cm diameter) or with shaking at 160 rpm [33] or on *Klebsiella aerogenes*-overlaid SM agar plates [34,35]. For antibiotic-resistant strains the AX2 medium [36] was either supplemented with 5 µg/mL G418 (Sigma-Aldrich, Darmstadt, Germany) or 5 µg/mL blasticidin S (ICN Biomedicals GmbH, Eschwege, Germany). Log phase cells (2–4 × 10^6^ cells/mL) were used for all experiments. The analysis of cell growth in shaking culture and on *K. aerogenes* as well as cell survival upon nitrogen starvation and development experiments were carried out as described [32,37]. Development was analyzed at specific time points using a stereomicroscope (M205 C, Leica, Wetzlar, Germany) and the accompanying Leica LAS X software (v.3.3.0). To assess spore viability, spore balls from one- to two-day old fruiting bodies were collected; the spores were then suspended in Soerensen’s phosphate buffer (2.0 mM Na_2_HPO_4_, 14.6 mM KH_2_PO_4_, pH 6.0) and their density adjusted to 2 × 10^4^ spores/mL. To compare spore viability of different *D. discoideum* strains, spores were either left untreated or subjected to 0.01% NP-40 or heat (45 °C, 30 min). 200 spores were plated together with *K. aerogenes* onto SM agar plates and incubated at 22 °C, and the number of colony forming units (CFUs) was determined after plaque appearance.

### 2.2. Vector Construction and Transformation

**Figure 2:**
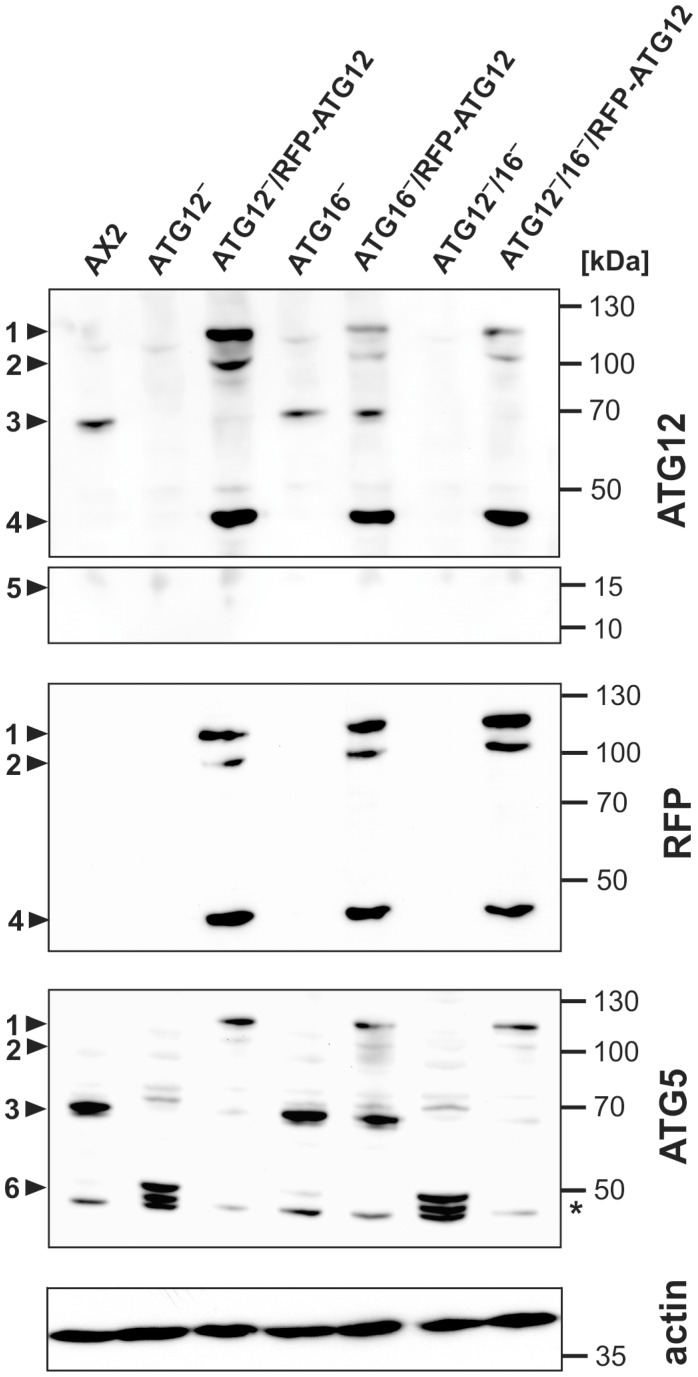
Verification of different mutant strains by immunoblotting of total cell lysates of wild-type AX2, ATG12‾, ATG16‾, and ATG12‾/16‾ cells, and of mutant strains that express RFP fused N-terminally to ATG12. The ATG12~ATG5 conjugate was detected at about 68 kDa in AX2, ATG16‾, and ATG16‾/RFP-ATG12 cell lysates, but not in the ATG12 knock-out strains (arrowhead 3). In knock-out strains expressing RFP-ATG12, the RFP and the ATG12 antibodies detected three bands (arrowheads 1, 2, and 4) and the ATG5 antibody detected two bands (arrowheads 1 and 2). No unconjugated ATG12 of about 14 kDa was detected in AX2 and ATG16‾ cells (arrowhead 5). Unconjugated ATG5 of about 46 kDa was detected in ATG12‾ and ATG12‾/16‾ cells (arrowhead 6). Arrowhead legend: 1 = 115 kDa; 2 = 103 kDa, RFP-ATG12~5; 3 = 68 kDa, ATG12~5; 4 = 46 kDa, RFP-ATG12; 5 = 14 kDa, position of unconjugated ATG12; 6 = 46 kDa, unconjugated ATG5. * = degradation product of ATG5. ATG12, RFP, and actin were visualized on the same membrane. ATG5 immunoblots were performed separately. Actin was used as a loading control. Top rows: ATG12 mAb; upper middle row: RFP pAb; lower middle row: ATG5 pAb; bottom row: actin mAb.

The *atg12* gene replacement construct was generated in the pLPBLP vector where the bsr resistance cassette is flanked by loxP sites [38]. A PCR-amplified 3′ fragment of 585 bp was cloned into the *Hin*dIII and *Sal*I sites and a 5′ fragment of 569 bp was cloned into into the *Pst*I and *Bam*HI sites of the pLPBLP vector (Figure S1A). The plasmid was introduced into AX2 cells and the ATG16‾ mutant [32] by electroporation, and transformants were selected in the presence of 5 µg/mL blasticidin S [39]. Gene replacement mutants were identified by PCR screening of blasticidin-resistant clones, followed by qRT-PCR, RNA_seq_ analysis and Western blotting (Figure 2 and Figure S1B–D). The bsr cassette was excised from the single and double knock-out strains through transient expression of the Cre recombinase as described [40] (Figure S1A). For ectopic expression of full-length ATG12 N-terminally tagged with RFP, the coding sequence of *atg12* was amplified by PCR and cloned into the p338-19 mRFPmars vector [41] via the *Bam*HI and *Eco*RI restriction sites. In the encoded RFP-ATG12 fusion protein a nine amino acids GGSGGSGGS linker sequence separates the RFP moiety from the full-length ATG12. For ectopic expression of RFP-GFP-ATG8a, the coding sequence of GFP was amplified by PCR and inserted via *Eco*RI into the previously described mRFPmars-ATG8a vector [42]. The final expression constructs were verified by sequencing. The plasmids were introduced into AX2 and the ATG12‾, ATG16‾, and ATG12‾/16‾ mutants by electroporation, and transformants were selected in the presence of 5 µg/mL G418. G418-resistant clones expressing RFP-ATG12 or RFP-GFP-ATG8a were identified by visual inspection under a fluorescence microscope (Nikon Eclipse Ts2-FL) and verified by Western blotting.

### 2.3. Antibody Generation, SDS-PAGE, and Western Blotting

For generation of ATG12-specific monoclonal antibodies (mAbs), the coding sequence of *atg12* was amplified by PCR and inserted into the pGEX-6P-1 expression vector (GE Healthcare GmbH, Solingen, Germany). The GST-ATG12 fusion protein was expressed in *E. coli* XL1 Blue, purified using Glutathione Sepharose^®^ 4B beads (GE Healthcare GmbH), released through cleavage with PreScissionTM protease (GE Healthcare GmbH), and used for immunization of female BALB/c mice which were 6–10 weeks of age, as described [43]. Hybridoma supernatants were screened for their ability to recognize the RFP-ATG12 fusion protein in Western blots of total cell lysates of the ATG12‾/[act15]:RFP-ATG12 strain. Mice were handled in accordance with the German Animal Welfare Act (Tierschutzgesetz) as well as the German regulation for the protection of animals used for experimental purposes or other scientific purposes (Tierschutz-Versuchstierverordnung), and the investigations were approved by the responsible governmental animal care and use office (Landesamt für Natur, Umwelt und Verbraucherschutz North Rhine-Westphalia, Recklinghausen, Germany; reference number 84-02.05.40.14.080).

SDS-PAGE and Western blotting of total cell lysates (2 × 10^5^ cells per lane) were performed as described [42,44,45]. The generated (K89-141-1) ATG12 mAb was used for Western blotting at a 1:2 dilution. GFP was detected with mAb K3-184-2 at a 1:50 dilution [46], RFP with a specific polyclonal antibody (pAb) at a 1:50,000 dilution [Jan Faix, unpublished], ATG5 with a specific pAb at a 1:500 dilution (Malte Karow, unpublished), actin with the mAb Act1-7 at a 1:40 dilution [47], the proteasomal subunit psmA7 (SU7) with the mAb 171-337-2 at a 1:50 dilution [48], and ubiquitin with the mAb P4D1 at a 1:1000 dilution (Cell Signaling Technology, Frankfurt, Germany). Secondary antibodies used were anti-mouse and anti-rabbit IgG conjugated to horseradish peroxidase (Sigma-Aldrich, Darmstadt, Germany) at a 1:10,000 dilution followed by chemiluminescence detection. Images were recorded using an Intas ECL Chemostar documentation system. LabImage 1D L-340 software (Intas Science Imaging Instruments GmbH, Göttingen, Germany) was used for signal quantification as well as for protein molecular weight determination.

### 2.4. RNA Sequencing Analysis

Total RNA from either vegetative or 3 or 6 h starved (in Soerensen’s phosphate buffer) *D. discoideum* cells was isolated as described [49], and RNA quality control was performed using the Agilent 2100 bioanalyzer (Agilent Technologies, Ratingen, Germany). Only RNA with an RNA Integrity Number (RIN) above eight was used for RNA library generation with the Ribo-Zero™ rRNA Removal Kit (Plant Seed/Root, Illumina Inc., San Diego, CA, USA). For the synthesis and amplification of cDNA prior to sequencing, the TruSeq Stranded Total RNA Library Prep Kit (Illumina Inc., USA) was utilized according to the manufacturer’s instructions. All cDNA samples were pooled in equivalent amounts and fragmented into smaller cDNAs with lengths of 200 to 500 bases, followed by 5′ and 3′ adapter ligation. Next generation sequencing was performed with a HiSeq run on an Illumina Genome Analyzer HiSeq 4000 machine. Obtained sequences were filtered and preprocessed as described [50], aligned to the AX4 reference genome [51], and evaluated using QuickNGS version 1.26 [50]. This analysis is based on two different methods, namely, the DESeq2 package and Cufflinks2 [52,53]. Based on our evaluation of the two normalization methods, we decided to use the DESeq2 data for further analysis [52]. The RNAseq raw data, fragments per kilobase of transcript per million mapped reads (FPKM) values and experimental information have been submitted to gene expression omnibus (GEO) (https://www.ncbi.nlm.nih.gov/geo/) and are available under the accession number GSE123780. Volcano-plots [54] were generated in the statistical software environment R (v.2.15.0, R Development Core Team, 2012) and RStudio (v.0.99.465, RStudio Team, 2015). Differentially regulated genes with a *p*-value ≤ 0.05 and an absolute fold change ≥ 2.0 were further analyzed. Venny 2.1 (http://bioinfogp.cnb.csic.es/tools/venny/index.html) was used for the generation of Venn diagrams and PANTHER version 11.1 [55] for gene ontology (GO) analysis. 

### 2.5. Fluorescence Microscopy

Immunofluorescence microscopy was essentially done as described [37]. Confluently grown *D. discoideum* cells were harvested from culture plates, washed in Soerensen’s phosphate buffer and 5.7 × 10^6^ cells were seeded into 6 cm Ø petri dishes containing round cover slips (Ø 12 mm). Cells were allowed to adhere for 15 min, after which the medium was carefully removed and cells were washed twice in Soerensen’s phosphate buffer and fixed with −20 °C cold methanol for 5 min. Fixed cells were washed three times with 1× PG buffer (1× PBS containing 10 mM glycine) for 5 min and blocked twice in 1× PBG buffer (1× PBS containing 0.5% BSA and 0.045% fish gelatin) for 15 min at room temperature. Fixed cells were incubated with the monoclonal P4D1 anti-ubiquitin antibody (Cell Signaling Technology, Frankfurt, Germany) at a 1:100 dilution in PBG buffer. The secondary antibody was Alexa-fluor 568 conjugated goat anti-mouse IgG at a 1:10,000 dilution (Invitrogen GmbH, Darmstadt, Germany). Nuclei were stained with 1 µg/mL 4′,6-diamidino-2-phenylindole (DAPI, Sigma-Aldrich, Darmstadt, Germany).

The RFP-GFP-ATG8a autolysosome maturation assay was essentially done as described [56]. Cells were resuspended at a density of 2 × 10^6^ cells/mL in Soerensen’s phosphate buffer, transferred to a µ-Dish (Ø 35 mm, ibidi GmbH, Munich, Germany) and allowed to settle down for 15 min. In case of ectopic GFP expression, cells were washed once with LoFlo (low fluorescence) medium (ForMedium™ Ltd., Hunstanton, UK) and incubated in the same medium overnight to reduce background fluorescence. The medium was removed and 1 mL Soerensen’s phosphate buffer was added for 2 h to induce autophagy by starvation. The autophagic flux was slowed down by incubation of the cells with Soerensen’s phosphate buffer containing 100 mM NH_4_Cl for 4 h. Images of fixed and live cells were taken with an inverted Leica TCS SP5 confocal laser scanning (Leica, Wetzlar, Germany) with a 100× HC PL APO 1.40 oil immersion objective. Excitation of DAPI was set at 405 nm and emission at 412–461 nm, excitation of GFP was set at 488 nm and emission at 500–540 nm, and excitation of RFP or Alexa-fluor was set at 568 at 568 nm and emission at 580–620 nm. Images were processed using the accompanying Leica LAS AF Lite software (v.1.3.0), Adobe Photoshop CS (v.8.0) and CorelDRAW^®^ 2017. Deconvolution of captured images was performed by means of the Huygens Essential software (v.16.05, Scientific Volume Imaging B.V.).

### 2.6. Proteasomal Activity Analysis

Proteasomal activity measurements of the different *D. discoideum* strains were conducted using the established protocol from skeletal muscle tissue [57] with minor changes as described [58]. Mean values and standard errors of three independent experiments were calculated. The chymotrypsin-like activity of AX2 wild-type cells was set to 1.

### 2.7. GFP Cleavage Assay

The proteolytic GFP cleavage assay to monitor autophagy in *D. discoideum* was conducted as described using AX2, ATG12‾, ATG16‾, and ATG12‾/16‾ cells expressing RFP-GFP-ATG8a [40]. The lysosomotropic compound NH_4_Cl was used to raise the lysosomal pH, thus allowing the accumulation of cleaved RFP-GFP derived from autophagic degradation of the expressed RFP-GFP-ATG8a fusion protein. Three independent experiments were performed.

### 2.8. Miscellaneous Methods

RNA isolation and cDNA generation were performed as described [49]. Quantitative phagocytosis of TRITC-labelled heat-killed yeast cells and macropinocytosis of TRITC-labelled dextran was conducted as outlined [32]. Mean values and standard errors of three independent experiments were calculated.

### 2.9. Statistics and Reproducibility

Unless otherwise indicated, all data shown are derived from at least three independent experiments, and mean values and the standard error of the mean (SEM) are depicted wherever applicable. The analysis of the statistical significance of experimentally detected differences was carried out with the software environment R (v.2.15.0, R Development Core Team, 2012). The Shapiro-Wilk test served as a test of normality and Levene’s test assessed the equality of variances for data. If the null-hypothesis was accepted for both tests, ANOVA was used as a parametric test and Tukey’s test as appropriate post hoc analysis. A rejected null-hypothesis led to the implementation of the non-parametric Kruskal-Wallis and the Dunn-Bonferroni tests as post hoc analysis. Three levels of significance were defined as follows: *p*-value: ≤ 0.05 = significant *; ≤ 0.01 = very significant **; ≤ 0.001 = extremely significant ***. Details are given in the figure legends. 

## 3. Results

### 3.1. ATG12 Is Evolutionarily Conserved

All ATG12 proteins contain a so-called APG12 domain, which has no explicit sequence homology to ubiquitin but shows the conserved ubiquitin-fold region in the crystal structure [11]. This domain, which spans amino acid 38 to 124 in *D. discoideum* ATG12, is required for both ATG12~5 conjugation and autophagy [19]. We performed multiple sequence alignments of the ATG12 orthologs from *D. discoideum*, *S. cerevisiae*, *Arabidopsis thaliana*, *Hydra vulgaris*, *Drosophila melanogaster*, *Danio rerio*, and *Homo sapiens*. The alignment revealed a number of amino acids, which are evolutionary highly conserved in ATG12 (Figure 1B). For instance, the C-terminal glycine residue that forms a covalent bond with an internal lysine of ATG5 is absolutely conserved in all aligned species [59]. The K38 residue, which mediates the interaction with ATG3, and the hydrophobic phenylalanine (F92 in *D. discoideum*), which is crucial for autophagosome formation, are also absolutely conserved [59]. Moreover, the D97 and C106 residues in *D. discoideum*, which comprise the E3 activity of the ATG12~5/16 complex, are conserved in all species except *S. cerevisiae* and *A. thaliana* [60]. Interestingly, the N-terminal region varies in both sequence and length among the ATG12 orthologs, indicating species-specific functions [19]. Alignment of the entire *D. discoideum* ATG12 protein sequence with the orthologs from the other species revealed sequence identities of 29% with *D. melanogaster*, 31% with *H. vulgaris*, 34% with *D. rerio,* 37% with *S. cerevisiae*, 37% with *H. sapiens*, and 44% with *A. thaliana*.

ATG12 is part of the ATG12~5/16 complex, which is indispensable for the expansion and closure of the isolation membrane [61]. To decipher the cellular function of ATG12, we generated gene replacement mutants of *atg12* in AX2 wild-type and ATG16‾ cells (Figure S1A). We confirmed the generated strains by genomic PCR, PCR of cDNA, qRT-PCR and RNA_seq_ analysis (Figure S1B–D and Figure 4C). We next generated strains that expressed RFP-ATG12 in the ATG12‾, ATG16‾, and ATG12‾/16‾ knock-out mutants. An overview of all strains used in this study is provided in Table 1. Western blot analysis of total cell homogenates from vegetative AX2 wild-type and mutant cells with the ATG12 mAb and the ATG5 pAb revealed only a single band of around 68 kDa in AX2 wild-type and ATG16‾ cells, which was missing in ATG12‾ and ATG12‾/16‾ cells (Figure 2, arrowhead 3). Since no unconjugated ATG12 of about 14 kDa (Figure 2, arrowhead 5) and of ATG5 of about 46 kDa (Figure 2, arrowhead 6) were detectable in AX2 wild-type and ATG16‾ cells, as evidenced by the ATG12 mAb and the ATG5 pAb, we conclude that conjugation of ATG12 to ATG5 is very efficient in vivo. Unconjugated ATG5 of about 46 kDa was detected in ATG12‾and ATG12‾/16‾ strains (Figure 2, arrowhead 6). In the ATG12‾, ATG16‾, and ATG12‾/16‾ strains expressing RFP-ATG12, the ATG12 mAb, the ATG5 pAb, and the RFP pAb detected three different protein species of about 115 kDa, 103 kDa, and 46 kDa (Figure 2, arrowheads 1, 2, and 4). Based on their calculated molecular masses, they are the unconjugated RFP-ATG12 fusion protein with 46 kDa, the RFP-ATG12~5 conjugate with 103 kDa, and possibly the RFP-ATG12 fusion protein covalently conjugated to ATG5 and ATG10 with 115 kDa. We assume that the detection of unconjugated RFP-ATG12 and of the RFP-ATG12~10~5 trimer is due to the overexpression of RFP-ATG12 in these strains. Alternatively, the RFP-ATG12~5 conjugate could migrate at 115 kDa, a higher molecular mass than expected, and the protein species running at 103 kDa could constitute a degradation product (Figure 2, arrowheads 1 and 2). No *Dictyostelium* ATG10 antibody is available to clarify this point.

### 3.2. Cellular Processes Dependent on Canonical Autophagy Are Severely Impaired in ATG12 and ATG16 Knock-Out Strains

**Figure 3:**
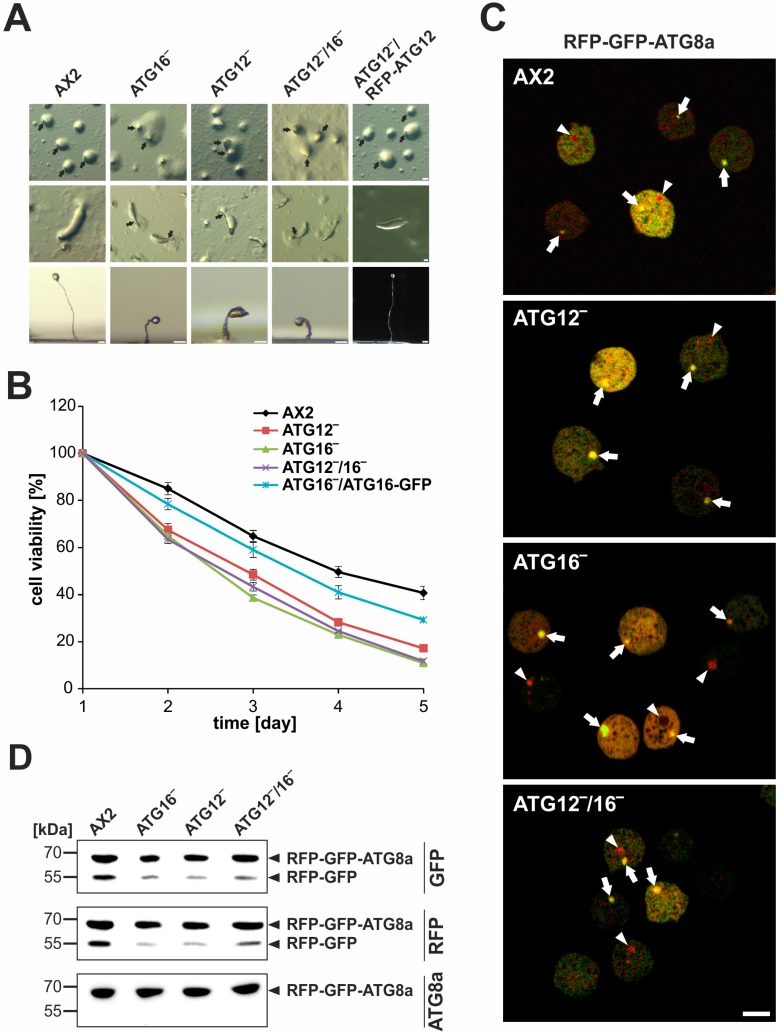
Development, cell survival upon nitrogen starvation, and autolysosome formation of AX2 and different knock-out strains. (**A**) Development of mutant strains on phosphate agar plates is severely impaired. Shown are three developmental stages. Top row: Top view of the tipped mound stage. Wild-type AX2 cells generated only one tip per mound while mutant strains generally produced two or three tips per mound (black arrows). Middle row: Top view of the slug stage. Slugs of mutants were thinner than AX2 slugs and frequently broke apart (black arrows). Bottom row: Side view of terminally differentiated fruiting bodies. In comparison to AX2 cells, the generated fruiting bodies of the mutants were much smaller (please note the different scale), were misshapen, and had a thickened stalk. Expression of RFP-ATG12 in the ATG12 mutant background completely rescued the developmental phenotype. Scale bar is 25 µm. (**B**) Cell survival of AX2 and mutant cells. The different strains were maintained in SIH medium without amino acids for five days. Cell survival was assessed by counting the number of colony-forming units (CFUs) on a lawn of *K. aerogenes* at the indicated times. CFUs at day 1 was set to 100% for each strain. The difference between AX2 and the knock-out strains was extremely significant from day 2 to day 5. For statistical analysis Tukey’s test was used as post hoc analysis. Mean values and standard errors of the mean (SEM) of three independent experiments are shown. (**C**) Analysis of autolysosome formation in AX2, ATG12‾, ATG16‾, and ATG12‾/16‾ cells expressing RFP-GFP-ATG8a. To visualize autophagosomes and autolysosomes, cells were treated with 100 mM NH_4_Cl for 4 h to slow down the autophagic flux and examined by live cell imaging. Red punctae (arrowheads) highlight autolysosomes and yellow punctae (arrows) indicate autophagosomes. The scale bar is 5 µm. See Table 2 for quantitative analysis. (**D**) Proteolytic cleavage assay to monitor autophagy. RFP-GFP-ATG8a was expressed in AX2, ATG12‾, ATG16‾, and ATG12‾/16‾ cells, respectively. Cells were treated two times for two hours with 100 mM NH_4_Cl, lysed, and total cell lysates were subjected to Western blotting. RFP-GFP-ATG8a and cleaved RFP-GFP were detected with a polyclonal ATG8a antibody, a GFP-specific monoclonal antibody, and an RFP-specific polyclonal antibody. As compared to AX2 significantly less RFP-GFP cleavage product was produced in the mutant strains. The positions of RFP-GFP-ATG8a and of RFP-GFP are indicated. No free GFP or RFP at around 26 kDa was detectable. A representative Western blot of three independent experiments is shown.

In response to starvation, *D. discoideum* cells enter development, which ultimately results in the formation of mature fruiting bodies. In this process, a significant part of the required energy for morphogenesis is mobilized by autophagy; thus, most autophagy-deficient mutants display abnormal development [27]. We found that development on phosphate agar plates of the ATG12‾ and ATG12‾/16‾ mutants was severely impaired in a manner similar to that previously reported for ATG9‾ and ATG16‾ mutants [32,37]. In contrast to AX2 wild-type cells, mutant strains usually generated tipped mounds with two or three tips per mound and their slugs were generally thinner and frequently broke apart. Furthermore, fruiting body formation of all mutant strains took considerably longer and the terminally differentiated fruiting bodies had a crippled shape with thickened stalks and were extremely tiny in comparison to AX2 wild-type fruiting bodies. There was no significant difference in the developmental phenotype of ATG12‾, ATG16‾, and ATG12‾/16‾ mutants, and expression of RFP-ATG12 in the ATG12‾ mutant background completely rescued the developmental phenotype (Figure 3A). The severe impairment of fruiting body formation of ATG12- and ATG16-deficient cells prompted us to analyze spore viability of spores from AX2, ATG12‾, ATG16‾, and ATG12‾/16‾ fruiting bodies. We observed a dramatic reduction of spore viability for untreated spores and for spores treated either with NP40 or heat from knock-out strains. Furthermore, we observed significant differences between the different knock-out strains, with lowest spore viability for the ATG12‾/16‾ strain, followed by the ATG16‾ and then the ATG12‾ strain (Figure S2).

Cell survival upon nitrogen starvation also strongly depends on autophagy and we next examined cell viability of the knock-out mutants in comparison to AX2 wild-type cells. AX2, ATG12‾, ATG16‾, and ATG12‾/16‾ cells as well as ATG16‾ cells expressing ATG16-GFP as a control were starved in an amino acid free medium and the number of CFUs was determined on a lawn of *Klebsiella aerogenes* every 24 h. After five days of nitrogen starvation, approximately 40% of AX2 and 30% of ATG16‾/ATG16-GFP cells were still viable. By contrast, less than 17% of the ATG12‾, ATG16‾, and ATG12‾/16‾ mutants had retained viability (Figure 3B).

To elucidate the involvement of ATG12 in autolysosome maturation, we expressed the autophagosomal marker protein ATG8a as RFP-GFP-ATG8a fusion protein in AX2, ATG12‾, ATG16‾, and ATG12‾/16‾ cells (Figure S3). Since the acidic environment of lysosomes quenches the fluorescence of GFP, autolysosomes appear red in fluorescence microscopy, while neutral autophagosomes appear yellow through the emission of red and green light from RFP and GFP, respectively [56]. Cells were incubated with NH_4_Cl to slow down the autophagic flux, and confocal microscopy of live cells was performed (Figure 3C). Quantification showed that in ATG12‾, ATG16‾, and ATG12‾/16‾ cells the percentage of autolysosomes was approximately three times lower than in AX2. In addition, we found a slight decrease in the average number of autophagic punctae per cell for ATG12‾, ATG16‾, and ATG12‾/16‾ cells (Table 2). Autophagic degradation of substrates can be monitored with the GFP cleavage assay [40]. This assay is based on the observation that the GFP moiety is often cleaved as a whole from GFP-tagged autophagic substrates inside the autolysosome and accumulates because of its resistance to further digestion. We expressed RFP-GFP-ATG8a in AX2, ATG12‾, ATG16‾, and ATG12‾/16‾ cells, respectively, and found that upon treatment with NH_4_Cl significantly less RFP-GFP cleavage product was produced in the mutant strains as compared to AX2 (Figure 3D). The results indicate that both core autophagy proteins, ATG12 and ATG16, contribute together as a functional unit to autophagosome maturation.

### 3.3. Autophagy Mutants Display Massive Transcriptional Changes

**Figure 4:**
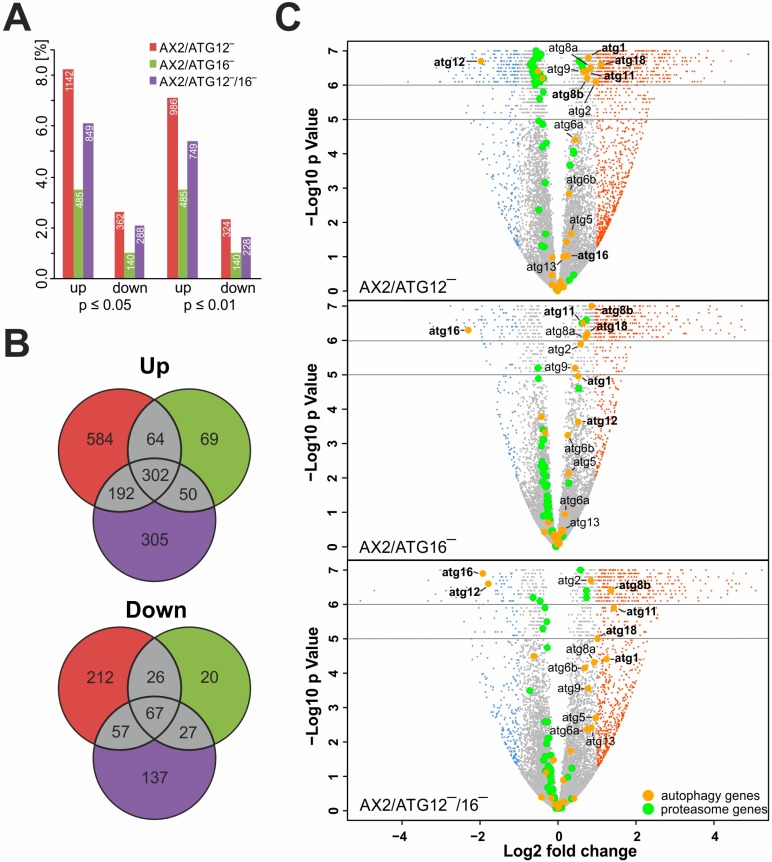
Differentially regulated genes in comparisons of AX2 with ATG12‾, ATG16‾, and ATG12‾/16‾ strains. (**A**) Percentage of up- and down-regulated genes in dependence of different thresholds for fold change (FC ≥ 2.0, ≤ 0.5) and *p*-value (*p* ≤ 0.05, ≤ 0.01) are shown. RNA was isolated from vegetative cells and six biological replicates of each strain were analyzed. Transcriptome: 13,866 transcription units. (**B**) Venn diagrams of differentially regulated genes of AX2 versus ATG12‾ (left circle, red), AX2 versus ATG16‾ (right circle, green), and AX2 versus ATG12‾/16‾ (lower circle, purple). Differentially regulated genes common for two or three comparisons are shown in grey. Top, up-regulated and bottom, down-regulated genes. Only those genes with fold change ≥2.0 or ≤0.5 and *p* ≤ 0.05 were used as input. (**C**) Presentation of differentially regulated genes in a volcano-plot for each strain comparison. Differentially regulated genes with Log2 fold change ≥1 and *p* ≤ 0.05 are labelled red, genes with Log2 fold change ≤−1 and *p* ≤ 0.05 are labelled blue, autophagy-related genes are highlighted by bigger orange-filled circles, and proteasome-related genes are highlighted by bigger green-filled circles. For better visualization, all genes with a *p*-value between 10^−5^ and 10^−6^ are randomly distributed in the area from 10^−5^ to 10^−6^. All genes with a *p*-value = 0 (i.e. values < 10^−6^) are randomly distributed in the area from 10^−6^ and 10^−7^. Autophagy genes with a fold change ≥2.0 or ≤ 0.5 and *p* ≤ 0.05 in at least one of the comparisons are indicated by their Demerec name. The plot was created using the R environment (v. 2.15.0).

We carried out RNA_seq_ analysis to determine transcriptional changes of ATG12‾, ATG16‾, and ATG12‾/16‾ cells in comparison to AX2. First, we compared the differential regulation of the entire transcriptome of the different mutant strains to AX2 in dependence of different thresholds for fold change and *p*-value. The analysis revealed for fold changes ≥ 2.0 and *p*-values ≤ 0.05 and ≤ 0.01 very high numbers of differentially regulated genes and the number of up-regulated genes was approximately two to three times higher than the number of down-regulated genes (Figure 4A). A decrease in the *p*-value from ≤ 0.05 to ≤ 0.01 only marginally decreased the number of differentially regulated genes, and we decided to use a fold change ≥ 2.0 and a *p*-value ≤ 0.05 for further analysis. With 8.2% (1142) up- and 2.6% (362) down-regulated genes the number of differentially regulated genes was highest for ATG12‾, intermediate for ATG12‾/16‾ (with 849 (6.1%) up- and 288 (2.1%) down-regulated genes) and lowest for ATG16‾ cells (with 485 (3.5%) up- and 140 (1.0%) down-regulated genes) (Figure 4A). The intermediate number of differentially regulated genes in the ATG12‾/16‾ double knock-out strain suggests opposite regulation for a subset of the genes in the ATG12‾ and the ATG16‾ strains. We further compared the up- and down-regulated genes of all three mutant strains graphically in Venn diagrams. The absolute number of genes common to either two strains was very similar for the ATG12‾ and ATG16‾ strains as well as for the ATG16‾ and ATG12‾/16‾ strains, while the ATG12‾ and ATG12‾/16‾ strains shared considerably more genes (Figure 4B). This result is even more evident if we look at the percentages of common differentially regulated genes (Table 3). It is of note that nearly 60% of the differentially regulated genes of ATG16‾ cells are shared with the ATG12‾ and ATG12‾/16‾ strains. In total, 1566 genes were up- and 546 were down-regulated in either one, two, or all three strains. Of these genes, the three strains had 302 (19.2%) of the up- and 67 (12.2%) of the down-regulated genes in common (Figure 4B). Next, we used volcano-plots to identify changes in our data sets by plotting statistical significance versus differential regulation [62]. In the generated plots for the three knock-out strains, significantly up- and down-regulated genes (fold change (FC) ≥ 2.0, *p*-value ≤ 0.05) are displayed as red and blue dots, respectively, and un-regulated genes as grey dots (Figure 4C). Two findings are obvious: (i) the much higher fraction of up-regulated genes in all three knock-out strains and (ii) the significantly higher number of differentially regulated genes in the ATG12‾ strain and the significantly lower number of differentially regulated genes in the ATG16‾ strain. We highlighted genes encoding core autophagy proteins (orange circles) and added the gene names for those genes that displayed a FC ≥ 1.5 and a *p*-value ≤ 0.05 in at least one of the knock-out strains (Figure 4C). Applying these parameters, we found eleven significantly up-regulated core autophagy genes. All these genes were differentially regulated in the ATG12‾/16‾ strain but only some of them in the other two strains. Since our autophagy mutants also displayed a significant reduction in proteasomal activity (see below) we also highlighted all genes encoding proteasomal subunits (Figure 4C, green dots). We found that 38 out of the 40 proteasomal genes were un-regulated and the remaining two were only slightly up-regulated (Table S1). Thus, the expression levels of all subunits of the proteasome were either unchanged or only marginally affected in the mutant strains.

### 3.4. ATG12 and ATG16 Deficiency Results in the Up-Regulation of Other Core Autophagy Genes

**Figure 5:**
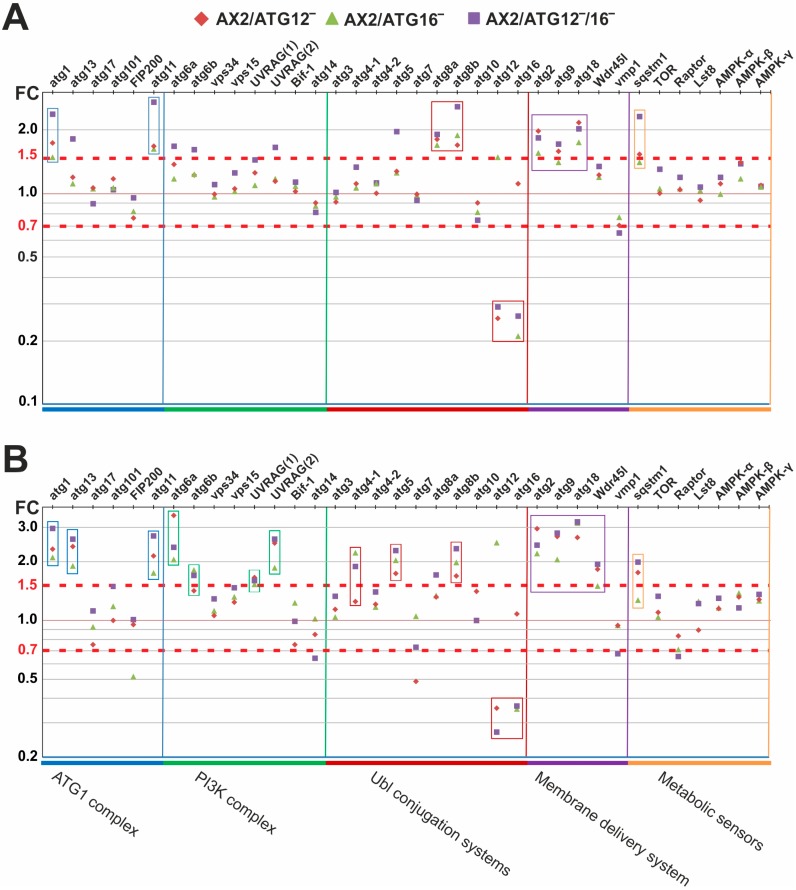
A subset of autophagy-related genes is up-regulated in ATG12‾, ATG16‾, and ATG12‾/16‾ cells. (**A**) RNA from vegetative cells was isolated and RNA_seq_ analysis was performed. All autophagy-related genes identified so far were assigned to functional categories. The threshold for up- and down-regulated genes was set to FC ≥ 1.5 or FC ≤ 0.7 and *p* ≤ 0.05. Six biological replicates of each strain were analyzed. (**B**) Upon starvation the up-regulation of several autophagy-related genes is more pronounced in ATG12‾, ATG16‾, and ATG12‾/16‾ cells. RNA from cells that were starved for 3 h (t3) was isolated and RNA_seq_ analysis was performed. Three biological replicates of each strain were analyzed.

Based on the results from the volcano-plot, we next evaluated specifically all genes encoding autophagy proteins from vegetative (t0) cells and from cells starved for 3 h (t3). The RNA_seq_ analysis revealed that the core autophagy genes *atg1*, *atg11*, *atg13*, *atg6a*, *atg6b*, *atg5*, *atg8a*, *atg8b*, *atg2*, *atg9*, and *atg18*, as well as the autophagy adaptor *sqstm1*, were up-regulated more than 1.5-fold in at least one of the mutant strains (Figure 5A; Table S2). The gene products are members of different functional classes of the autophagy system, namely, the ATG1 complex, the PI3K complex, the ubiquitin-like conjugation systems, the autophagosomal membrane delivery system, and the adaptors and regulators. Their notable up-regulation, including in particular the enrichment of genes encoding proteins involved in the early phase of autophagy, indicates the existence of a sensing system for autophagosome formation. Since autophagy is induced in response to starvation, we also analyzed the differential regulation of ATG12‾, ATG16‾, and ATG12‾/16‾ cells, which were starved for 3 h (t3) in comparison to starved AX2 cells. The analysis revealed more up-regulated genes in each functional class in these strains, and their up-regulation was more pronounced than in vegetative cells (Figure 5B). The RNA_seq_ analysis of autophagy-related genes also confirmed the knock-out mutants, since *atg12* and *atg16* were strongly down-regulated in their respective strains under vegetative and starved conditions (Figure 5). To decipher enriched biological processes, molecular functions, and cellular components in the differentially regulated gene sets of autophagy-deficient strains, we performed gene ontology (GO) analysis [55]. For the biological process domain we found that genes encoding components of sporulation, signal transduction, metabolic processes, transmembrane transport, and, as expected, macroautophagy, were enriched among the up-regulated genes of all three mutant strains. These results are in perfect agreement with respect to the severe developmental phenotypes of the autophagy compromised strains. Among the down-regulated genes we found that among others the biological process categories endocytosis, phagocytosis, and cell motility were enriched (Table 4; a full list of all enriched biological processes, molecular functions, and cellular component categories is provided in Table S3).

### 3.5. Endocytosis Is Significantly Impaired in Mutant Strains

**Figure 6:**
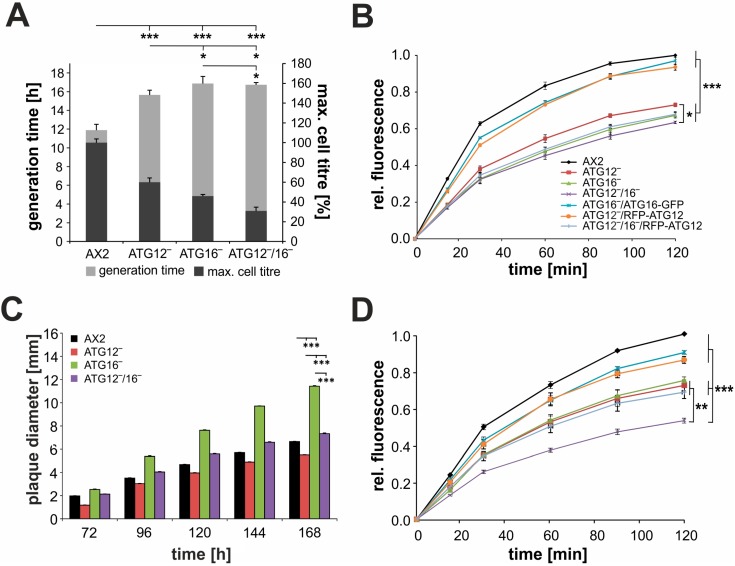
Analysis of endocytosis in AX2 and mutant strains. (**A**) Generation time and maximal cell titre of AX2, ATG12‾, ATG16‾, and ATG12‾/16‾ cells in shaking culture. The *p*-values refer to the difference in maximal cell titre. (**B**) Macropinocytosis of TRITC-labelled dextran. The analyzed *Dictyostelium* strains were adjusted to 6 × 10^6^ cells/mL and TRITC-dextran was added at a final concentration of 2 mg/mL. Macropinocytosis was measured as relative fluorescence for 2 h at the indicated time points. The final fluorescence of AX2 was set to 1. (**C**) Growth of AX2, ATG12‾, ATG16‾, and ATG12‾/16‾ cells on a lawn of *K. aerogenes*. Plaque diameters were quantified every 24 h from day 3 until day 7 after plating of *D. discoideum* cells. Mean values and SEM of 100 plaques are depicted. For statistical analysis the Dunn-Bonferroni test, implemented in R as post hoc analysis, was performed. Statistical significance between AX2 and the different knock-out strains is depicted for the 168 h time point. (**D**) Phagocytosis of TRITC-labelled yeast is impaired in mutant strains. Cells were resuspended at 4 × 10^6^ cells/mL in Soerensen’s phosphate buffer and fluorescent yeast cells were added in a six-fold excess. Phagocytosis was determined as relative fluorescence for 2 h at different time points. The final fluorescence of AX2 was set to 1. The same color code as in (B) was used. For statistical analysis in A, B, and D Tukey’s test, implemented in R, was used as post hoc analysis, and mean values and SEM of three independent experiments were calculated. *** *p* ≤ 0.001; ** *p* ≤ 0.01; * *p* ≤ 0.05.

Based on the results from GO analysis, we analyzed endocytosis in more detail. At first, we assessed cell growth in shaking culture, since the axenic strains of *Dictyostelium* are capable of consuming liquid nutrients by macropinocytosis [63]. We found that generation times in the logarithmic growth phase were increased by about 40% for the different knock-out strains. In addition, the maximum cell titre was significantly decreased by about 40% for ATG12‾, 54% for ATG16‾, and 69% for ATG12‾/16‾ cells in comparison to AX2 (Figure 6A). These defects could be caused by less efficient macropinocytosis and/or a deficiency in the intracellular utilization of nutrients. Therefore, we next analyzed the macropinocytic uptake of TRITC-labelled dextran. All three mutant strains showed similar significantly reduced pinocytic activity. At the 2 h time point the relative fluorescence of ATG12‾, ATG16‾, and ATG12‾/16‾ cells was 27%, 33%, and 37%, respectively, lower than for AX2 (Figure 6B). We also found a slightly significant difference (*p*-value ≤ 0.05) in the uptake activity of the ATG12‾ and ATG12‾/16‾ strains at later time points. Expression of ATG16-GFP in the ATG16‾ strain and of RFP-ATG12 in the ATG12‾ strain rescued the reduced pinocytosis defect (Figure 6B). As expected, the expression of RFP-ATG12 in the ATG12‾/16‾ mutant was not sufficient to rescue the pinocytosis defect. These results support a defect in the uptake of nutrients in the ATG12‾, ATG16‾, and ATG12‾/16‾ cells. 

Next, we analyzed growth on a bacterial lawn of *K. aerogenes*, where an initially single *Dictyostelium* cell clears the bacteria by phagocytosis [64,65]. Tiny plaques become apparent on the bacterial lawn around 72 h after spreading the cells, and the diameter of the plaques then increased steadily over the next few days. We found that plaque diameters were significantly larger for ATG16‾ and smaller for ATG12‾ cells in comparison to AX2. Plaque diameters of ATG12‾/16‾ were intermediate-sized and similar to those of AX2 (Figure 6C and Figure S4). This result suggests that ATG12 and ATG16 have opposite functions in this process. To analyze phagocytosis more directly, we next investigated phagocytosis of TRITC-labelled yeast. The overall kinetics, with an almost linear increase in fluorescence in the initial phase followed by a progressively more subtle increase in the remaining time of the assay, was similar for all strains. However, in comparison to AX2, the ATG12‾/RFP-ATG12, and the ATG16‾/ATG16-GFP cells, the increase in fluorescence of the ATG12‾, ATG16‾, and ATG12‾/16‾ strains was slower and final fluorescence values were significantly lower. In addition, the defect in phagocytic activity was nearly identical for ATG12‾, ATG16‾, and ATG12‾/16‾/RFP-ATG12 cells, while it was more pronounced in the ATG12‾/16‾ double knock-out strain (Figure 6). It appears, therefore, that both ATG12 and ATG16 have independent functions in the phagocytosis of yeast or that the absence of ATG12 or ATG16 from the ATG12~5/16 complex may not fully inhibit the activity of the complex. We conclude that the autophagy mutants behave differently with respect to phagocytosis of yeast and the clearance of *K. aerogenes*.

### 3.6. Protein Homeostasis Is Disturbed in Mutant Strains

**Figure 7:**
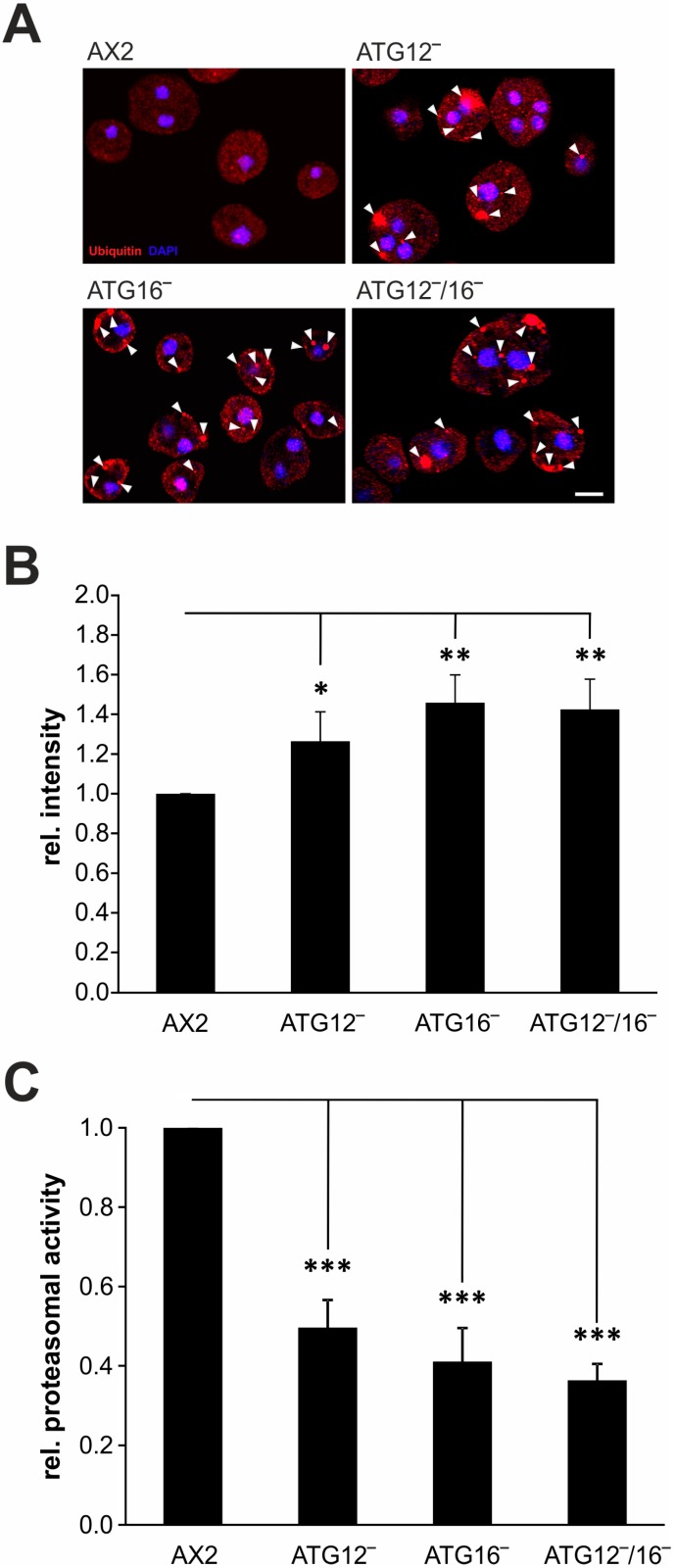
Protein homeostasis is disturbed in the knock-out strains. (**A**) Immunofluorescence microscopy of AX2, ATG12‾, ATG16‾, and ATG12‾/16‾ cells. Cells were fixed with cold methanol and stained with the mAb P4D1. Large ubiquitin-positive protein aggregates in mutant strains are marked by arrowheads. Nuclei were visualized by DAPI staining. The scale bar is 5 µm. (**B**) Quantification of global protein ubiquitination. The P4D1 signal intensities of five blots were quantified and normalized with the actin signal. The signal intensity of AX2 was set to 1. For statistical analysis the Dunn-Bonferroni test was used as post hoc analysis. ** *p* ≤ 0.01; * *p* ≤ 0.05. (**C**) Proteasomal activity of AX2 and knock-out strains. The proteasomal activity assay was conducted as described [32]. The specific proteasomal activity was calculated by normalization with the amount of proteasomal subunit psmA7 (SU7) expression as shown in Figure S5B. The chymotrypsin-like activity of AX2 was set to 1. Three independent experiments were performed and mean values and SEM calculated. For statistical analysis Tukey’s test was used as post hoc analysis. *** *p* ≤ 0.001.

Autophagy and the ubiquitin-proteasome system (UPS) are both critical pathways for protein degradation in eukaryotic cells and are thus mainly responsible for protein homeostasis. In addition, there is increasing evidence for crosstalk between both systems [30,32,66,67,68,69]. First, we conducted confocal microscopy of fixed cells which had been stained with the P4D1 anti-ubiquitin antibody. In comparison to AX2 cells, we detected many large ubiquitin-positive protein aggregates in ATG12‾, ATG16‾, and ATG12‾/16‾ cells (Figure 7A). Next, we examined the level of ubiquitinated proteins in total cell lysates of AX2 wild-type, ATG12‾, ATG16‾, and ATG12‾/16‾ cells. Quantification of Western blots revealed increases of 26%, 46%, and 43% in the amount of ubiquitinated proteins for ATG12‾, ATG16‾, and ATG12‾/16‾ cells, respectively (Figure 7B and Figure S5A).

The increase in ubiquitinated proteins in ATG12‾, ATG16‾, and ATG12‾/16‾ cells suggests an imbalance in protein homeostasis which could, at least in part, be counteracted by the UPS. This could be achieved by an increase in the number of proteasomes and/or an up-regulation of proteasomal activity. We first analyzed proteasome abundance. The RNA_seq_ results showed that the expression of nearly all proteasomal genes was unchanged in the mutant strains (Figure 4C). Only the genes encoding psmB1 and psmD14 were slightly up-regulated (Table S1). Western blot analysis also revealed that the protein level of psmA7 (SU7), as representative of the 20S proteasome, was unchanged in ATG12‾, ATG16‾, and ATG12‾/16‾ strains (Figure S5B). For determination of the specific proteasomal activity we used the supernatant after lysis of the cells and centrifugation (Figure S5C). The analysis revealed a strong reduction to 50%, 41%, and 36% in ATG12‾, ATG16‾, and ATG12‾/16‾ cells, respectively, in comparison to AX2 (Figure 7C). Taken together, autophagy deficiency led to a decrease in the proteasomal activity without apparently influencing proteasomal number. Thus, contrary to expectations, the UPS is not capable of compensating a defect in autophagy. It is actually dependent on intact autophagy for full activity.

## 4. Discussion

### 4.1. Unconjugated ATG12 Is not Detectable in Total D. Discoideum Cell Lysates

In an analogous fashion to classic ubiquitination, the ubiquitin-like protein ATG12 is transferred from the E1-like enzyme ATG7 via the E2-like protein ATG10 to ATG5, and the ATG12~5 conjugate is formed [11] (Figure 1A). In total cell lysates of either vegetative or starved AX2 wild-type and ATG16‾ cells we could only detect the ATG12~5 conjugate with an apparent molecular mass of about 68 kDa, but never the unconjugated ATG12 monomer of about 14 kDa (Figure 2). In addition, unconjugated ATG5 of about 46 kDa was only detectable in the ATG12‾and ATG12‾/16‾ strains (Figure 2, arrowhead 6). Hence, we conclude that conjugation of ATG12 to ATG5 is very efficient *in vivo*. Unconjugated ATG12 has so far only been detected in *A. thaliana* and in U2OS cells, where the estimated half-life was 30 min [70,71]. Treatment of U2OS cells with the proteasome inhibitor MG132 increased the level of unconjugated ATG12, indicating that monomeric ATG12 is rapidly degraded by the UPS [71]. In ATG7-, ATG3- and ATG5-deficient mouse embryonic fibroblasts (MEFs) no ATG12~5 conjugate can be generated; nevertheless, the level of unconjugated ATG12 was unchanged in comparison to wild-type MEFs despite an up-regulation of the *atg12* mRNA [71]. These results can be explained by a substantial difference in the stability between free ATG12 and the ATG12~5 conjugate, which is not fully understood. An attractive possibility is that ATG5 masks a destabilizing region in ATG12 [71,72]. In the ATG12~5 conjugate, both proteins are oriented to each other in such a manner that conserved residues on each molecule form a continuous stable patch, which results in stabilization of ATG12 [60]. 

In the ATG12‾ and ATG16‾ strains expressing RFP-ATG12, we were able to detect additional bands that likely represent the RFP-ATG12~10~5 intermediate and the RFP-ATG12 fusion protein besides the expected RFP-ATG12~5 conjugate (Figure 2, arrowheads 1, 2, and 4). Since ATG12 becomes covalently linked to ATG7, ATG10, and ATG5 in the course of the ubiquitin-like protein conjugation system, the presence of further ATG12 conjugates in Western blot analysis is, per se, not surprising [11]. However, unconjugated ATG12 and the ATG12~10~5 intermediate were not detectable in AX2 wild-type and ATG16‾ cells. Fusion of RFP or other tags with target proteins may interfere with post-translational modification, protein folding, stability, function, structure, and protein-protein interactions [73]. The appearance of additional bands suggests that the fusion of RFP to the N terminus of ATG12 somehow interferes with the conjugation reactions and also influences the stability of the ATG12 monomer.

### 4.2. Massive Transcriptional Changes Occur in Mutant Strains

Several RNA_seq_ studies have already provided a snapshot of actively expressed transcripts under different conditions in *D. discoideum*. Since the social amoebae enter a developmental program upon starvation, the developmentally regulated, protein-coding transcriptome has been characterized extensively [74,75,76,77]. Even the long noncoding RNA transcriptome of *D. discoideum* has recently been analyzed [78]. However, no global survey of the transcriptome in autophagy-deficient strains of *D. discoideum* has been performed so far. Our RNA*seq* analysis of ATG12‾, ATG16‾, and ATG12‾/16‾ cells revealed major transcriptional changes in a large number of genes in comparison to AX2 (Figure 4A). ATG12‾ cells shared a higher percentage of up- and down-regulated genes with ATG12‾/16‾ than with ATG16‾ cells (Figure 4B; Table 3). On the other hand, more than 50% of the differentially regulated genes of ATG16‾ cells were in common with the other two knock-out strains. The intermediate number of differentially regulated genes in the ATG12‾/16‾ strain (lower than in ATG12‾ but higher than in ATG16‾) suggests opposite regulation for a subset of the genes in the ATG12‾ and ATG16‾ strains. We also found that there is a much higher fraction of up- than down-regulated genes in all three knock-out strains (Figure 4C). GO analysis revealed several enriched categories in ATG12- and ATG16-deficient strains and underlined the importance of autophagy for the maintenance of cellular homeostasis within an organism (Table 4, Table S2).

We specifically investigated whether the absence of ATG12, ATG16, or both proteins influences the transcription of other core autophagy genes whose gene products are involved in autophagosome formation. We found that *atg1*, *atg11*, *atg8a*, *atg8b*, *atg2*, *atg9*, and *atg18*, as well as the autophagy adaptor *sqstm1*, were up-regulated more than 1.5-fold in at least one of the mutant strains (Figure 5A). As expected, the up-regulation of autophagy-related genes was more pronounced in starved cells (t3) [79] (Figure 5B). In mammalian cells, the up-regulation of core autophagy genes is induced by transcription factors of the FoxO family [80,81]. Taken together, the significant up-regulation of a number of core autophagy genes in the autophagy-deficient mutants indicates the existence of a sensing system for autophagosome formation, which co-ordinately regulates the abundance of the involved ATG proteins [32].

### 4.3. Protein Homeostasis Is Impaired in ATG12- and ATG16-Deficient Strains

Until recently, the UPS and autophagy were considered two independent protein degradation machineries with no point of interaction, since both systems have different substrate preferences and separate molecular mechanisms. However, evidence is accumulating which indicates that these two pathways, which are of utmost importance for the clearance and recycling of cellular material, are somehow interrelated [30,66,67,69,82,83]. Currently, more than 40 proteins are known to be shared as either substrates or regulators of both autophagy and the UPS, among them core autophagy proteins like ATG5, ATG7, ATG8, and ATG16 [83,84,85,86,87]. Several studies have reported compensatory up-regulation of autophagy upon inhibition of proteasomal activity [67,88,89]. This notwithstanding, the effect of autophagy deficiency on the activity of the UPS is controversial [90,91]. We found that ablation of ATG12 or ATG16 resulted in a strong imbalance in protein homeostasis, as evidenced by an increase in ubiquitinated proteins, the appearance of ubiquitin-positive protein aggregates, and a decline in proteasomal activity (Figure 7 and Figure S5). Proteasomal activity was about 50% lower in ATG12‾ cells in comparison to AX2 and slightly further decreased in ATG16‾ and ATG12‾/16‾ cells (Figure 7C). Concomitant with decreased proteasomal activity the amount of ubiquitinated proteins significantly increased in the mutant strains (Figure 7B and Figure S5A). An increase in the amount of ubiquitinated proteins in *D. discoideum* appears to be a general consequence of the impaired autophagic activity, as this has already been reported for ATG1‾, ATG5‾, ATG7‾, ATG9‾, ATG9‾/16‾, ATG8a‾, and ATG8a‾/b‾ cells [29,32,37,58,68]. The defect in autophagy could in principle be counteracted by the UPS through either an increase in the number of proteasomes and/or an up-regulation of proteasomal activity. However, in our analysis the proteasomal activity of all autophagy-deficient strains was reduced, and the number of proteasomes, as evidenced by the quantification of the proteasomal subunit psmA7, was unchanged (Figure 7C and Figure S5B). In agreement with this result our RNA_seq_ analysis showed that the mRNA levels of 38 out of the 40 proteasomal genes were unchanged and the remaining two were only slightly up-regulated in the mutant strains (Figure 4C; Table S1). Thus, contrary to expectations, the UPS cannot compensate for a defect in autophagy. It is actually dependent on intact autophagy for full activity in *D. discoideum*.

Recently, proteaphagy as a novel type of selective autophagy has been described for *S. cerevisiae* and *A. thaliana* [92,93,94]. It is believed that proteaphagy is responsible for the degradation of non-functional proteasomes. Thus, in autophagy-deficient strains non-functional proteasomes would accumulate and hence result in the observed decline in proteasomal activity in ATG12‾, ATG16‾, and ATG12‾/16‾ cells. Interestingly, in mammals the half-life and cellular concentration of ATG12 and ATG16 themselves appear to be regulated by the UPS [71,95], and in *D. discoideum* ATG16 directly interacts with PSMD1 and PSMD2, two subunits of the 19S regulatory particle, and mediates their degradation via autophagy [87].

### 4.4. ATG12 and ATG16 Are Required for Efficient Autolysosome Maturation

The formation of a complete autophagosome is dependent on membrane extension, for which two ubiquitin-like conjugation machineries are required [11]. In the first ubiquitin-like reaction, ATG12 is covalently attached to ATG5 and subsequently two ATG12~5 conjugates interact with an ATG16 dimer to form a hetero-tetrameric complex. This complex promotes the conjugation of ATG8 to PE of the autophagic membrane in the last reaction of the second ubiquitin-like system [18,20]. Since ATG12 and ATG16 together with ATG5 fulfil a key role in membrane elongation of the phagophore, we assessed autolysosome maturation in the ATG12‾, ATG16‾, and ATG12‾/16‾ strains by expressing the autophagosomal marker protein ATG8a as RFP-GFP-ATG8a fusion protein (Figure 3C). We found that autolysosome maturation is similarly impaired in the three mutant strains with three times fewer autolysosomes as compared to AX2 (Table 2). In agreement with this result, significantly less RFP-GFP cleavage product released from RFP-GFP-ATG8a was detectable in a proteolytic cleavage assay [40] in the ATG12‾, ATG16‾, and ATG12‾/16‾ strains as compared to AX2 (Figure 3D). The absence of either ATG12 or ATG16 or both proteins makes autolysosome maturation more inefficient but does not completely block it. Autolysosome formation also still occurs, albeit with less efficiency, in ATG9‾ and ATG9‾/16‾ knock-out strains [32]. In line with our results it has recently been reported that in MEFs the two ubiquitin-like ATG conjugation systems are not essential for autolysosome formation but are important for efficient degradation of the inner autophagosomal membrane [61].

### 4.5. ATG12 and ATG16 Deficiency Causes Distinct Phenotypes

The importance of the ATG12~5/16 complex is illustrated by the fact that ATG12- or ATG5-deficient mice die one day after birth [24,96]. Deletion of the core autophagy genes *atg1*, *atg5*, *atg6*, *atg7*, *atg8a*, *atg8b*, *atg9*, and *atg16* in *D. discoideum* resulted in phenotypes of varying severity [23,32,37,68,97]. These results can be explained by either differences in the importance of the respective proteins for the functioning of autophagy and/or additional non-autophagic functions for some of these proteins. Indeed, in recent years more and more non-autophagic functions of core autophagy proteins have been reported [24,83,98]. Our results revealed complex phenotypes of different severity for the ATG12‾, ATG16‾, and ATG12‾/16‾ strains. We could distinguish four types: (i) no difference between the two single and the double knock-out (KO) strains, (ii) an increase in the severity of the phenotype from ATG12‾ to ATG16‾ but no further increase for the double KO, (iii) an incremental increase in the severity of the phenotype from ATG12‾ to ATG16‾ to the double KO, and (iv) opposing phenotypes (Table 5).

We observed type (i) for cellular processes, which fully depend on a functioning autophagy. The developmental phenotypes of the ATG12‾, ATG16‾, and ATG12‾/16‾ strains were similar to those previously reported for ATG5‾, ATG7‾, and ATG16‾ mutants [23,32]. Loss of either of these proteins led to severe impairments in the tipped mound stage, in the slug stage, and in fruiting body formation (Figure 3A). We also found similarly severe defects in autolysosome formation and cellular viability in response to amino acid starvation for the ATG12‾, ATG16‾, and ATG12‾/16‾ mutant strains (Figure 3B,C). These results imply that ATG12 and ATG16 act together with ATG5 as a functional unit in canonical autophagy. This is consistent with the similar phenotypes of ATG5‾ and ATG7‾ mutants which act upstream of the hetero-tetrameric ATG12~5/16 complex in the ubiquitin-like conjugation reaction [11,23,32]. In contrast, we observed a slightly more severe defect of ATG16‾ cells in comparison to ATG12‾ cells in proteasomal activity, axenic growth, and macropinocytosis (type (ii), Figure 6A,B and Figure 7C). In these cellular processes, ATG16 either fulfils an additional function or there is still some ATG5/16 complex with residual activity formed in the absence of ATG12. We recently found that ATG16 directly interacts with PSMD1 and PSMD2, two subunits of the 19S regulatory particle, which could explain the more severe defects of ATG16‾ cells in proteasomal activity [87]. The observed more severe defects of ATG16‾ cells in macropinocytosis, as evidenced by their reduced growth in liquid medium and reduced uptake of TRITC-labelled dextran, could be caused by a disturbance of recycling endosomes. It has been shown in HeLa cells that ATG9 and ATG16 also localize to recycling endosomes and that endosomal membranes likely contribute to autophagosome formation [99]. There is also a direct link between macropinocytosis and autophagy, since PtdIns3P signaling is also required for functional macropinocytosis [27,100]. Type (iii), that is, an incremental increase in the severity of the phenotype from ATG12‾ to ATG16‾ cells to the double KO, was the case for spore viability and maximal cell titre in liquid culture (Figure 6A and Figure S2). This suggests that both proteins fulfil an independent function in these cellular processes or that the ATG12~5/16 complex without either ATG12 or ATG16 has still some residual activity. Several autophagy-independent roles for ATG12 have already been described. Under certain nutrient limiting conditions, ATG12 interacts with ATG3 in maintaining mitochondrial homeostasis and preventing cell death [101,102]. Furthermore, ATG12 is involved in endosomal trafficking and IFNγ-mediated host defense against murine norovirus (MNV) infection in HeLa cells [103,104,105].

The results of the clearing assay for *K. aerogenes* revealed a decrease in plaque diameter for ATG12‾, an increase for ATG16‾, and an intermediate size for ATG12‾/16‾ cells (Figure 6C and Figure S4). This suggests that ATG16 has an inhibitory and ATG12 a stimulatory effect on the clearing of *K. aerogenes* (Table 5, type (iv)). Where exactly both proteins in this process act needs further investigation, because the differences in plaque size between the mutant strains could be caused not only by differences in phagocytosis activity, but also by differences in cell motility, bacterial sensing, intracellular killing of bacteria, metabolic adaptation, or even cell division [64]. In contrast, phagocytosis of TRITC-labelled yeast revealed similar defects for the ATG12‾ and ATG16‾ strains and a more severe defect in the double KO (Figure 6D). The exact link between autophagy and phagocytosis is currently not clear. LC3-associated phagocytosis (LAP) has been described in several systems [106]. There is general agreement that LAP requires the ATG12~5/16 complex for LC3 recruitment [107]. In addition, disruption of cellular recycling processes in autophagy-deficient strains could result in a shortage of membranes, which are essential for efficient phagocytosis. The observed differences in phagocytosis of yeast and *K. aerogenes* (Figure 6C,D) could also be caused by differences in microbial sensing and/or intracellular killing, as *Dictyostelium* uses distinct pathways to discriminate between different types of bacteria and other microorganisms [108,109]. Further analyses are needed to unravel the basis of these striking differences in the phagocytosis activity of the different autophagy-deficient strains.

## Figures and Tables

**Table 1:** *D. discoideum* strains used in this study.

Strains	Summary	References
ATG12‾	ATG12 null mutant	This work
ATG16‾	ATG16 null mutant	[32]
ATG12‾/16‾	ATG12/16 double null mutant	This work
ATG16‾/[act15]:ATG16-GFP	* Ect. exp. of ATG16-GFP in ATG16‾	[32]
ATG12‾/[act15]:RFP-ATG12	Ect. exp. of RFP-ATG12 in ATG12‾	This work
ATG16‾/[act15]:RFP-ATG12	Ect. exp. of RFP-ATG12 in ATG16‾	This work
ATG12‾/16‾/[act15]:RFP-ATG12	Ect. exp. of RFP-ATG12 in ATG12‾/16‾	This work
AX2/[act15]:RFP-GFP-ATG8a	Ect. exp. of RFP-GFP-ATG8a in AX2	[32]
ATG12‾/[act15]:RFP-GFP-ATG8a	Ect. exp. of RFP-GFP-ATG8a in ATG12‾	This work
ATG16‾/[act15]:RFP-GFP-ATG8a	Ect. exp. of RFP-GFP-ATG8a in ATG16‾	[32]
ATG12‾/16‾/[act15]:RFP-GFP-ATG8a	Ect. exp. of RFP-GFP-ATG8a in ATG12‾/16‾	This work

* Ect. exp.: ectopic expression.

**Table 2:** Autolysosome maturation is less efficient in ATG12‾, ATG16‾, and ATG12‾/16‾ cells. Statistical analysis was performed with the Dunn Bonferroni test implemented in R as post hoc analysis. The difference in the percentage of red punctae between NH_4_Cl-treated AX2 and the different knock-out strains is extremely significant. #, number.

Strain	Cells #	Punctae #	Punctae/Cell	Yellow Punctae #	Red Punctae		*p*-Value
					#	%	
AX2	300	419	1.40 ± 0.58	245	174	41.5 ± 2.0	
ATG12‾	300	396	1.32 ± 0.48	343	53	13.4 ± 1.0	≤ 0.001
ATG16‾	300	347	1.16 ± 0.36	304	43	12.4 ± 0.9	≤ 0.001
ATG12‾/16‾	300	364	1.21 ± 0.41	312	52	14.3 ± 0.7	≤ 0.001

**Table 3:** Common differentially regulated genes of ATG12‾, ATG16‾, and ATG12‾/16‾ cells. An absolute fold change of ≥2.0 and a *p* ≤ 0.05 was used for analysis. #, number.

	Strain	# of Genes	Common with ATG12‾		Common with ATG16‾		Common with ATG12‾/16‾		Common with the Other Two Strains
			#	%	#	%	#	%	%
Up	ATG12‾	1142			366	32	494	43	26
	ATG16‾	485	366	76			352	73	62
	ATG12‾/16‾	849	494	58	352	42			36
Down	ATG12‾	362			93	26	124	34	19
	ATG16‾	140	93	66			94	67	48
	ATG12‾/16‾	288	124	43	94	33			23

**Table 4:** Gene ontology (GO) term enrichment analysis of differentially regulated genes in ATG12‾, ATG16‾, and ATG12‾/16‾ cells. Selected enriched categories of the biological process for the up- and down-regulated gene sets that were common to all three mutant strains are listed. GO analysis was performed with PANTHER version 11.1 using genes with FC ≥ 2.0 or FC ≤ 0.5 and *p*-value ≤ 0.05 as input. The full list of enriched categories is provided in Table S2.

Enriched Biological Processes	
Up-Regulated Genes	Down-Regulated Genes
sporulation	phagocytosis
signal transduction	cell motility
cAMP-mediated signaling	endocytosis
macroautophagy	phototaxis
transmembrane transport	hyperosmotic response
metabolic process	

**Table 5:** Summary of the analyzed cellular processes in AX2, ATG12‾, ATG16‾, and ATG12‾/16‾ cells. Plus signs indicate an increase and minus signs a decrease in the corresponding cellular processes in comparison to AX2 cells. An increase in the number of plus or minus signs correlates with the severity of the respective defect.

Type	Cellular Process	Strains		
		ATG12‾	ATG16‾	ATG12‾/16‾
(i)	Development	−	−	−
	Autolysosome formation	−	−	−
	Cell viability	−	−	−
(ii)	Growth in shaking culture	−	− −	− −
	Macropinocytosis	−	− −	− −
	Proteasomal activity	−	− −	− −
(iii)	Spore viability	−	− −	− − −
	Maximal cell titre	−	− −	− − −
	Phagocytosis of yeast	−	−	− −
(iv)	Growth on *K. aerogenes*	−	+ +	+/−

## Supplementary Materials

The following supplementary information is available online http://www.mdpi.com/2073-4409/8/1/72/s1. Figure S1: Generation and verification of the *atg12* gene replacement mutant in AX2 and ATG16‾ cells, Figure S2: Spore viability was significantly reduced in ATG12‾, ATG16‾ and ATG12‾/16‾ cells, Figure S3: Western blot analysis of AX2 and mutant strains expressing RFP-GFP-ATG8a, Figure S4: Growth of AX2, ATG12‾, ATG16‾ and ATG12‾/16‾ cells on a lawn of *K. aerogenes*, Figure S5: Western blot analysis of global protein ubiquitination and proteasomal subunit psmA7 (SU7) expression in AX2 and mutant strains, Table S1: List of transcriptional regulation of proteasomal genes, Table S2: List of differentially regulated autophagosomal genes in vegetative cells, Table S3: Gene ontology (GO) term enrichment analysis of differentially regulated genes in ATG12‾, ATG16‾ and ATG12‾/16‾ cells.
