# Supplementary material for: Functional Characterization of Ubiquitin-Like Core Autophagy Protein ATG12 in Dictyostelium discoideum
Source: Cells. 2019 Jan 19;8(1):72. doi: 10.3390/cells8010072 (PMC6356199; doi:10.3390/cells8010072)
Supplement: Supplementary file 1 [file cells-08-00072-s001.pdf]

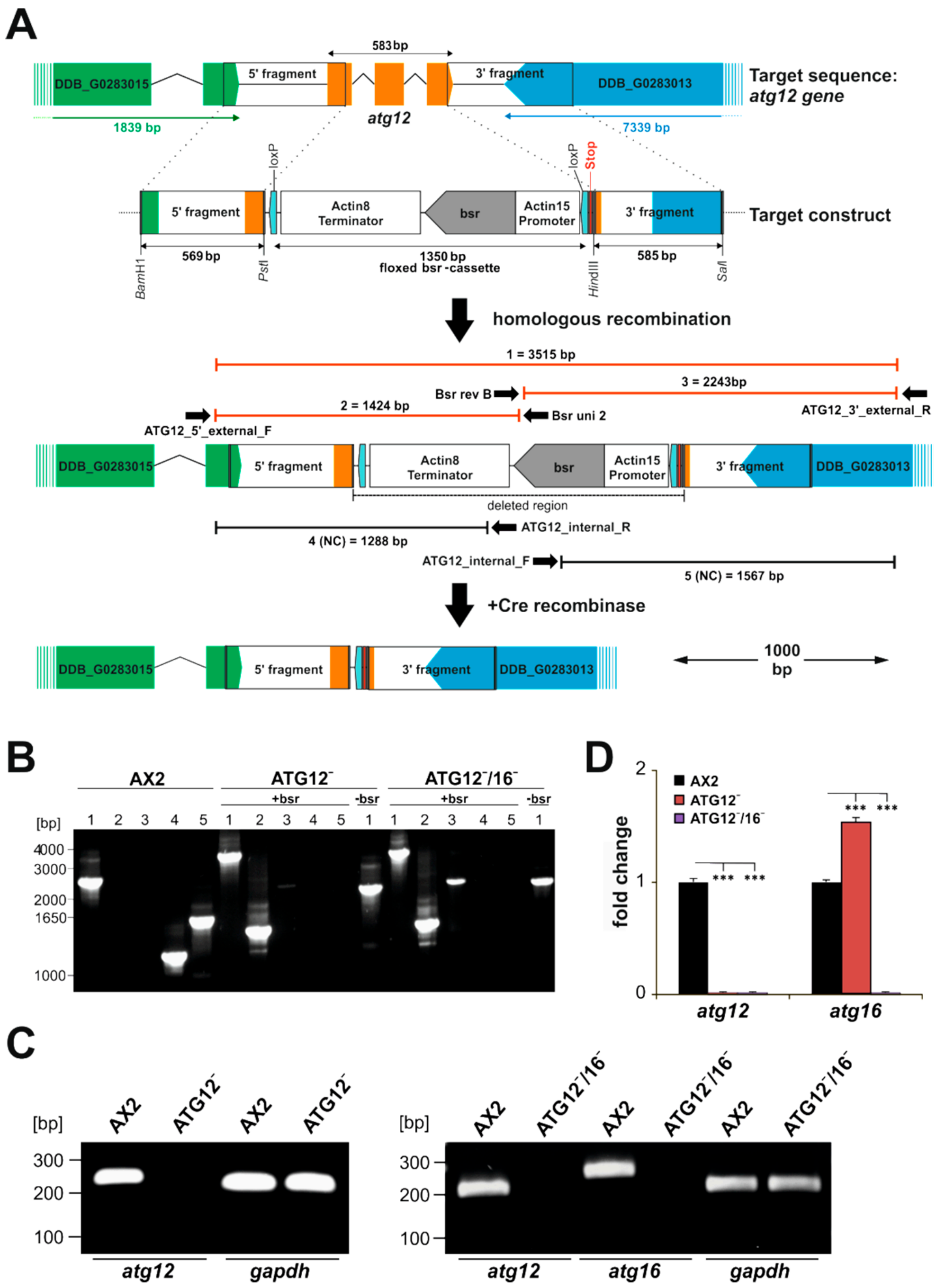

Figure S1. Generation and verification of the *atg12* gene replacement mutant in AX2 and ATG16⁻ cells. (**A**) The ATG12⁻ and ATG12⁻/16⁻ strains were generated by replacement of the *atg12* gene (583 bp) with the targeting construct containing the blasticidin resistance (bsr) cassette flanked by loxP sites. From the resulting ATG12 knock-out strains, the blasticidin cassette was removed by transient expression of the Cre recombinase. Restriction sites used for vector construction were: *Bam*HI, *Pst*I, *Hin*dIII and *Sal*I. The primer combinations 1–5 that were used for knock-out verification are shown. The 3′ ends of the neighboring genes of *atg12*, DDB_G0283015 (green) and DDB_G0283013 (blue), are schematically depicted. Gene orientation from 5′ to 3′ is indicated by the direction of the arrowheads. PCR product sizes are not drawn to scale. Introns are shown as a line. NC = negative control. (**B**) PCR confirmation of gene replacement in ATG12 knock-out strains. PCR with genomic DNA from AX2 wild-type cells served as control. Primer combinations used and expected product sizes are illustrated in (A). (**C**) and (**D**) qRT-PCR confirmation of ATG12 knock-out strains. Total RNA was isolated, reverse transcribed into cDNA and amplified with gene specific primers. *gapdh* was used as positive control and served for data normalisation. Expression of the respective cDNA in AX2 was set to 1. The Dunn-Bonferroni test was implemented in R as post hoc analysis. Mean values and SEM of three independent experiments are shown. ***, p-value ≤ 0.001.

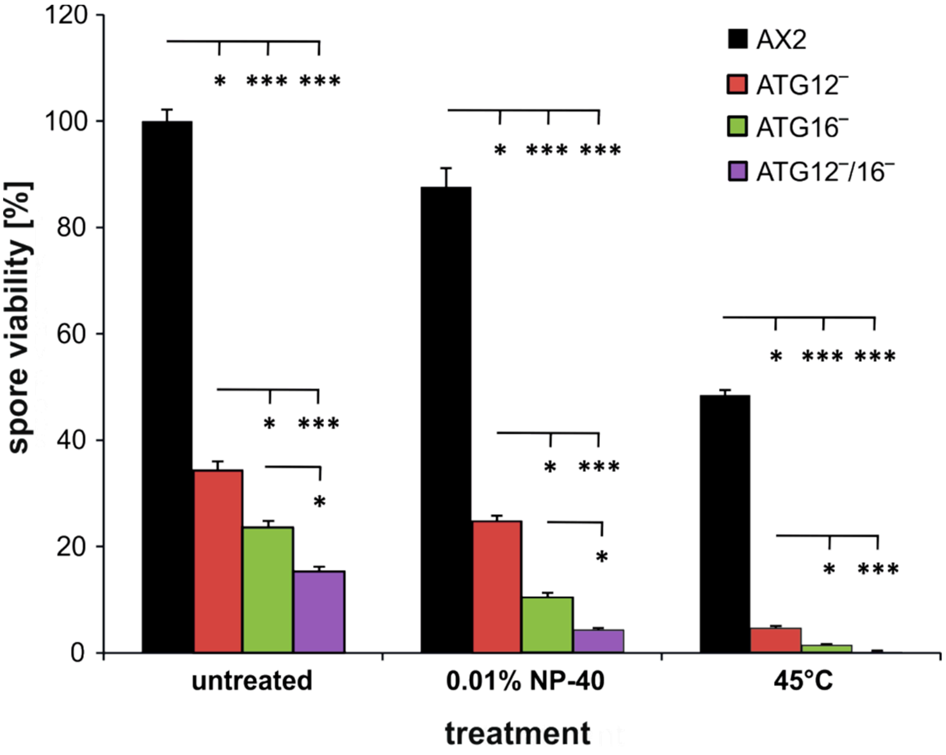

Figure S2. Spore viability was significantly reduced in ATG12^{-}, ATG16^{-} and ATG12^{-}/16^{-} cells. Spores of AX2 and mutant strains were plated on a lawn of *K. aerogenes* and plaques were enumerated after 3 days. Spores from the knock-out mutants, either untreated or treated with 0.01% NP-40 or heat at 45°C for 30 min, showed dramatically reduced spore viability. Spore viability of untreated AX2 spores was set to 100%. For statistical analysis the Dunn-Bonferroni test, implemented in R as post hoc analysis, was performed. Mean values and SEM of three independent experiments are shown. ***, p-value ≤ 0.001; *, p-value ≤ 0.05.

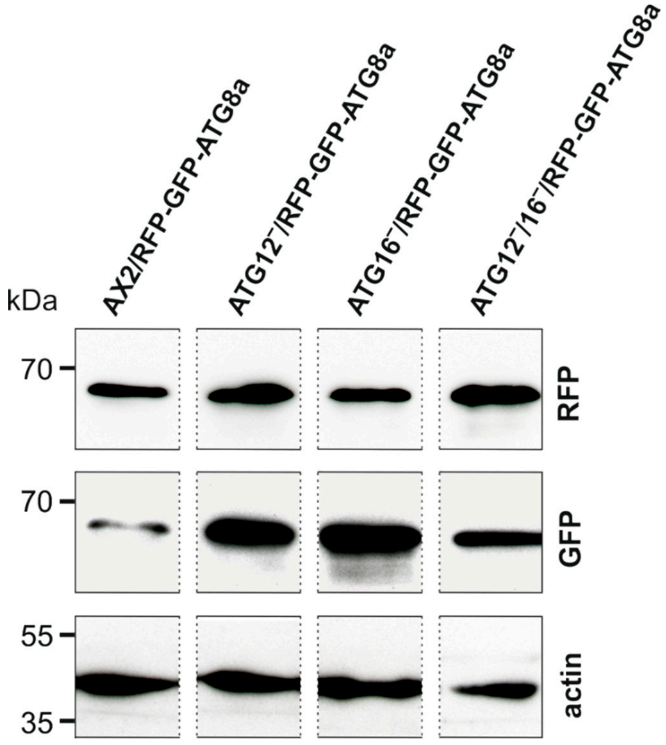

Figure S3. Western blot analysis of AX2 and mutant strains expressing RFP-GFP-ATG8a. Total cell lysates of AX2 and the different knock-out strains expressing RFP-GFP-ATG8a were probed with antibodies against RFP and GFP. Actin was detected with the mAb Act1-7 and served as loading control.

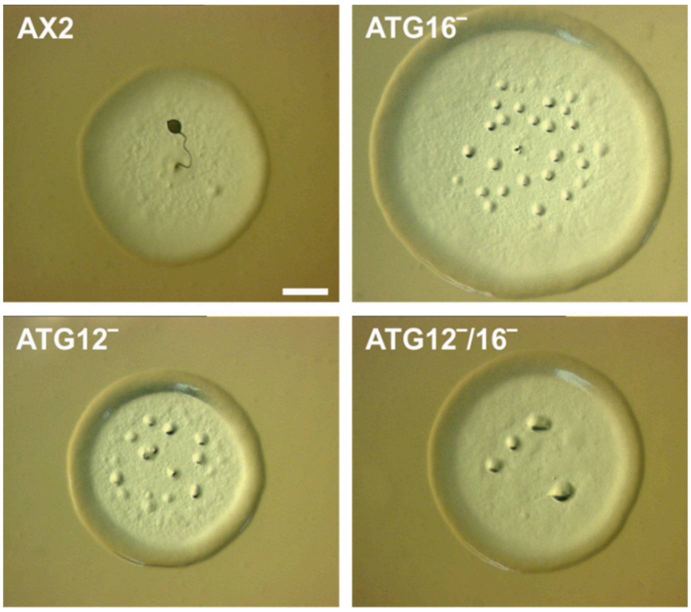

Figure S4. Growth of AX2, ATG12$^-$, ATG16$^-$ and ATG12$^-$/16$^-$ cells on a lawn of *K. aerogenes*. Representative images of single plaques after 96 h of growth on the bacterial lawn are shown. Scale bar is 500 µm.

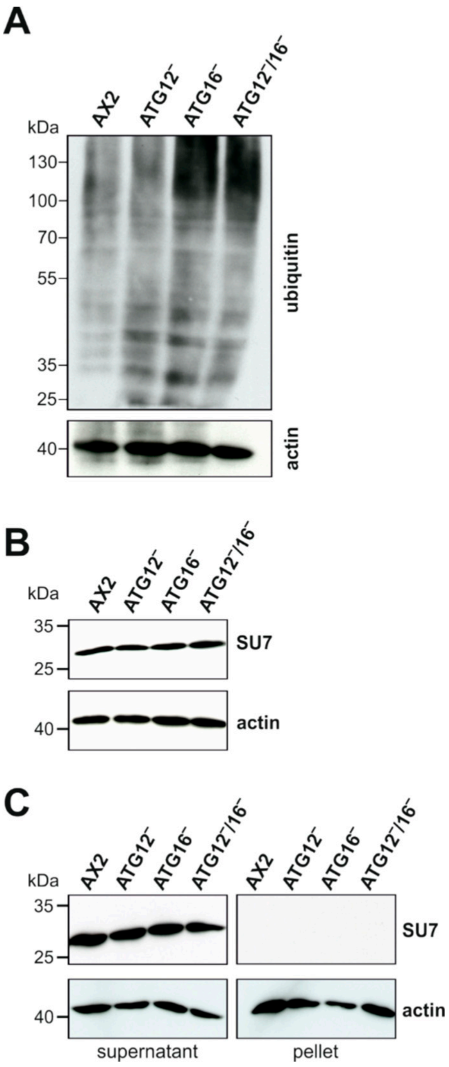

Figure S5. Western blot analysis of global protein ubiquitination and proteasomal subunit psmA7 (SU7) expression in AX2 and mutant strains. (**A**) A representative Western blot of total cell lysates using the mAb P4D1

is shown. (**B**) Western blot analysis of SU7 expression with the mAb 171-337-2 in total cell lysates of AX2 and mutant strains. (**C**) Western blot analysis of soluble and pelleted SU7 in AX2 and mutant strains. Cells were lysed and the lysate centrifuged as described for the proteasomal activity assay [32]. SU7 was exclusively found in the supernatant. Actin was always used as a loading control and detected with the mAb Act1-7.

Table S1. List of transcriptional regulation of proteasomal genes. Fold changes (FC) and p-values from six biological replicates subjected to RNA$_{seq}$ analysis were determined. None of the genes was more than 2-fold differentially regulated and only two genes were more than 1.5-fold differentially regulated in all three strains (highlighted in bold).

**Gene name**	**DDB_G ID**	**ATG12^{-}**		**ATG16^{-}**		**ATG12^{-}/16^{-}**	
		**FC**	**p-value**	**FC**	**p-value**	**FC**	**p-value**
psmA1	DDB_G0282363	0.71	0.00	0.86	0.08	0.84	0.05
psmA2	DDB_G0292122	0.78	0.00	0.88	0.12	0.83	0.00
psmA3	DDB_G0267408	0.66	0.00	0.85	0.06	0.85	0.08
psmA4	DDB_G0280969	0.68	0.00	0.85	0.02	0.76	0.00
psmA5	DDB_G0268538	0.68	0.00	0.81	0.00	0.82	0.00
psmA6	DDB_G0278847	0.71	0.00	0.82	0.00	0.83	0.00
psmA7	DDB_G0272831	1.46	0.00	1.49	0.00	1.49	0.00
**psmB1**	**DDB_G0272969**	**1.54**	**0.00**	**1.71**	**0.00**	**1.66**	**0.00**
psmB2	DDB_G0269472	0.66	0.00	0.86	0.04	0.79	0.00
psmB3	DDB_G0269772	0.78	0.00	0.93	0.35	0.91	0.25
psmB4	DDB_G0273163	1.23	0.48	1.13	0.49	1.07	0.82
psmB5	DDB_G0293784	0.64	0.00	0.77	0.00	0.79	0.00
psmB6	DDB_G0267390	0.65	0.00	0.93	0.38	0.88	0.02
psmB7	DDB_G0283697	0.88	0.07	0.95	0.43	1.03	0.68
psmC1	DDB_G0270784	0.74	0.00	0.87	0.05	0.96	0.65
psmC2	DDB_G0276917	0.72	0.00	0.85	0.05	0.94	0.46
psmC3	DDB_G0284415	0.77	0.00	0.85	0.03	0.88	0.07
psmC4	DDB_G0289003	0.68	0.00	0.80	0.01	0.83	0.01
psmC5	DDB_G0292382	0.80	0.00	0.87	0.07	0.94	0.41
psmC6	DDB_G0284517	0.75	0.00	0.88	0.07	0.88	0.14
psmD1	DDB_G0287953	0.68	0.00	0.80	0.01	0.92	0.24
psmD10	DDB_G0289189	1.32	0.00	1.25	0.01	1.19	0.11
psmD11	DDB_G0281315	0.58	0.00	0.79	0.00	0.79	0.03
psmD12	DDB_G0281051	0.61	0.00	0.72	0.00	0.82	0.01
psmD13	DDB_G0285105	0.67	0.00	0.76	0.00	0.78	0.03
**psmD14**	**DDB_G0272566**	**1.56**	**0.00**	**1.58**	**0.00**	**1.66**	**0.00**
psmD2	DDB_G0293752	0.71	0.00	0.83	0.02	0.98	0.84
psmD3	DDB_G0288621	0.65	0.00	0.73	0.00	0.73	0.00
psmD4	DDB_G0275755	0.98	0.79	0.93	0.15	0.76	0.00
psmD6	DDB_G0270188	0.76	0.00	0.81	0.01	0.87	0.28
psmD7	DDB_G0279633	0.82	0.00	0.89	0.16	1.02	0.86
psmD8	DDB_G0272564	1.33	0.34	0.99	0.96	1.27	0.45
psmD9	DDB_G0275753	1.24	0.00	0.79	0.01	0.64	0.00
psmE3	DDB_G0285099	0.79	0.05	0.84	0.12	0.61	0.00
psmE4	DDB_G0292398	1.01	0.92	0.94	0.35	1.02	0.78
psmF1	DDB_G0282617	1.33	0.00	1.06	0.48	1.28	0.06
psmG1	DDB_G0279769	0.75	0.05	0.80	0.13	0.83	0.21

psmG2	DDB_G0274447	0.71	0.00	0.78	0.00	0.82	0.07
psmG3	DDB_G0268522	0.98	0.90	1.00	0.99	0.96	0.84
psmG4	DDB_G0304543	0.81	0.02	0.86	0.10	0.80	0.07

Table S2. List of differentially regulated autophagosomal genes in vegetative cells. Fold changes (FC) and p-values from six biological replicates subjected to RNA_{seq} analysis were determined. Only those genes that were at least 1.5-fold differentially regulated in any of the three strains are depicted. FC values ≥ 2 or ≤ 0.5 are highlighted in bold and values ≥ 1.5 or ≤ 0.67 in italic.

DDB_G	Name	AX2/ATG12¯		AX2/ATG16¯		AX2/ATG12¯/16¯	
		FC	p-value	FC	p-value	FC	p-value
DDB_G0292390	*atg1*	*1.73*	0.00	1.48	0.00	**2.37**	0.00
DDB_G0277419	*atg2*	*1.97*	0.00	*1.55*	0.00	*1.83*	0.00
DDB_G0289881	*atg5*	1.27	0.02	1.25	0.01	*1.96*	0.00
DDB_G0269244	*atg6A*	1.37	0.00	1.17	0.11	*1.67*	0.00
DDB_G0288021	*atg6B*	1.22	0.00	1.23	0.00	*1.61*	0.00
DDB_G0286191	*atg8a*	*1.80*	0.00	*1.69*	0.00	*1.90*	0.00
DDB_G0290491	*atg8b*	*1.69*	0.00	*1.88*	0.00	**2.57**	0.00
DDB_G0285323	*atg9*	*1.58*	0.00	1.40	0.00	*1.71*	0.00
DDB_G0285767	*atg11*	*1.67*	0.00	*1.62*	0.00	**2.70**	0.00
DDB_G0282929	*atg12*	**0.26**	0.00	1.48	0.00	**0.28**	0.00
DDB_G0269192	*atg13*	1.19	0.09	1.11	0.32	*1.81*	0.00
DDB_G0275323	*atg16*	1.11	0.10	**0.21**	0.00	**0.26**	0.00
DDB_G0285375	*atg18*	**2.16**	0.00	*1.74*	0.00	*2.02*	0.00

Table S3. Gene ontology (GO) term enrichment analysis of differentially regulated genes in ATG12¯, ATG16¯ and ATG12¯/16¯ cells. RNA from vegetative cells was isolated and RNA_{seq} analysis was performed based on DESeq2. GO analysis was performed with PANTHER version 11.1 using genes with FC values ≥ 2 or ≤ 0.5 and a p value ≤ 0.05 as input. The listed enriched categories for the up- and down-regulated gene sets are common to all three mutant strains. Six biological replicates of each strain were analysed.

Biological process	Molecular function	Cellular component
	**Up-regulated enriched gene sets**	
sorocarp development	cAMP binding	plasma membrane
sorocarp morphogenesis	ATP binding	endosome membrane
spore wall assembly	catalytic activity	golgi membrane
sporulation	ATPase activity	ER membrane
cAMP-mediated signalling	protein binding	vacuole
macroautophagy	transporter activity	spore wall
gluconeogenesis	protein tyrosine/serine/threonine phosphatase activity	integral component of membrane
transport	G-protein coupled receptor activity	cytoplasmic vesicle
transmembrane transport	zinc ion binding	
cytolysis	hydrolase activity	
metabolic process		
signal transduction		
	**Down-regulated enriched gene sets**	
phagocytosis	structural constituent of cytoskeleton	phagocytic vesicle
cell motility	actin binding	phagocytic cup
endocytosis	actin filament binding	early phagosome
phototaxis	myosin binding	actin filament
cell morphogenesis	carbohydrate binding	actin cytoskeleton
hyperosmotic response		
